# A New Rapid Indirect ELISA Test for Serological Diagnosis of Feline Immunodeficiency

**DOI:** 10.3390/vetsci12020089

**Published:** 2025-01-23

**Authors:** Irene Ferrero, Paolo Poletti, Enrica Giachino, Joel Filipe, Paola Dall’Ara

**Affiliations:** 1 Agrolabo S.p.A., 10100 Scarmagno, Italy; 2 Department of Veterinary Medicine and Animal Sciences, University of Milan, 26900 Lodi, Italy

**Keywords:** FIV, immunodeficiency, FIVCHECK Ab ELISA, gp40, ELISA

## Abstract

The Feline Immunodeficiency Virus (FIV) is a lentivirus belonging to Retroviridae family that affects feline immune cells, causing a progressive immunosuppression by depleting CD4+ T-lymphocytes, similarly to the acquired immune deficiency syndrome (AIDS) in humans caused by human immunodeficiency virus (HIV). Diagnosis is usually performed by clinicians using rapid Enzyme-Linked Immunosorbent Assay (ELISA) or lateral flow tests that detect FIV antibodies. The aim of this work was the development of FIVCHECK Ab ELISA, a new rapid indirect assay for the detection of FIV antibodies in feline serum/plasma samples; FIVCHECK Ab ELISA was developed after a meticulous set-up and cut-off analysis through several methods, including the Youden’s index and ROC curve, to achieve the best test performance. The new kit was validated by testing 115 feline sera (38 positives and 77 negatives for FIV antibodies) against the ELISA rapid test SNAP FIV/FeLV Combo (IDEXX). Moreover, 103 sera (28 positives and 75 negatives) were also analyzed with two other rapid indirect ELISAss, INgezim FIV (Gold Standard Diagnostics) and VetLine FIV (NovaTec); FIVCHECK Ab ELISA agreed at 100% with SNAP (100% sensitivity, 95% confidence interval (CI): 88.5–100%; 100% specificity, 95% CI: 94.0–100%), 100% with INgezim FIV and 92.2% with VetLine FIV. Intra- and inter-assay accuracy and precision gave coefficients of variation lower than 10%. The new ELISA is a simple and quick test that provides reliable results for veterinary clinics and practices.

## Simple Summary

The aim of this work was the development of a new rapid indirect Enzyme-Linked Immunosorbent Assay (ELISA) assay, called FIVCHECK Ab ELISA, for the detection of antibodies against Feline Immunodeficiency Virus (FIV) in feline serum/plasma samples. FIV is a virus belonging to the Retroviridae family that affects feline immune cells, causing a progressive immunosuppression similar to the acquired immune deficiency syndrome (AIDS) in humans caused by human immunodeficiency virus (HIV). Diagnosis is usually performed by clinicians using rapid ELISA or lateral flow tests that detect FIV antibodies. The new test is the result of an extensive set-up phase carried out by the Research and Development (R&D) team of Agrolabo S.p.A., considering different concentrations of samples and reagents. In addition, the test cut-off was assessed and confirmed through several methods, including the Youden’s index and ROC curve, to achieve the best possible test performance in terms of sensitivity, specificity, accuracy, reliability, and repeatability of results. This study demonstrates that the FIVCHECK Ab ELISA has high sensitivity and specificity, making it also suitable for the analysis of highly lipemic or hemolytic samples, as no interferences were observed on the tested samples. In fact, FIVCHECK Ab ELISA was validated against the ELISA rapid test SNAP FIV/FeLV Combo (IDEXX) considered as reference: it agreed at 100%, with 100% sensitivity (95% confidence interval (CI): 88.5–100%) and 100% specificity (95% CI: 94.0–100%). Moreover, the performance of the new ELISA was compared with two other rapid indirect ELISAs widely used in veterinary practice, INgezim FIV (Gold Standard Diagnostics) and VetLine FIV (NovaTec). The FIVCHECK Ab ELISA agreed at 100% with INgezim FIV and 92.2% with VetLine FIV. Moreover, the new FIVCHECK Ab ELISA is an accurate and precise test with coefficients of variation lower than 10% both in intra- and inter-assays. The new test is fast as it provides correct and reliable results very quickly (25 min), without the need of particular laboratory equipment such as thermostats, because all incubations are performed at room temperature. Only the interpretation of results is performed instrumentally, by reading the OD values at 450 nm using an ELISA microplate reader (Multiskan SkyHigh, Thermo Fisher Scientific, Waltham, MA, USA). The new assay contains ready-to-use and safe reagents for the end-user and has a long-term storage up to 18 months at +2–8 °C. All these features make this test optimal for use in veterinary clinics and practices.

## 1. Introduction

The Feline Immunodeficiency Virus (FIV) is an enveloped single-stranded RNA lentivirus that belongs to the Retroviridae family isolated in cats [1]. FIV has many similarities to human immunodeficiency virus (HIV) and causes an AIDS-like syndrome in the domestic cat. FIV infects T-lymphocytes, cells of monocyte/macrophages/microglia lineage, and cells of the central nervous system. The viral replication is initiated by the interaction of the viral glycoproteins with CD134, up-regulated on activated CD4+ T cells, which permits the virus entry into the host cell cytoplasm [2]. FIV integrates its viral retrotranscribed RNA into DNA in the cat’s genome.

The integrated provirus is bordered by long terminal repeats (LTRs) and possesses *gag*, *pol*, and *env* genes, common elements of all retroviruses, and several genes that code for small accessory proteins such as Vif and Rev. The *gag* gene codes for a myristoylated matrix p15 protein, a capsid p24, and a nucleocapsid p13 proteins. FIV *pol* codes for the protease, reverse transcriptase, integrase, and deoxyuridine triphosphatase. The *env* gene codes for the heavily glycosylated surface unit protein gp95 and the transmembrane protein gp40 [2,3]. Three phases of FIV infection are recognized: primary (acute), subclinical, and clinical. In the first phase, the animal is viremic, and a sharp decline in lymphocyte populations, particularly CD4+ (helper) T-lymphocytes, occurs. In the second and longest phase, cats enter a long subclinical phase that can last for many years; in this case, the level of circulating free virus is suppressed, viral replication is very limited, the production of FIV antibodies is persistently high and the animal is clinically healthy. The levels of CD8+ T-lymphocytes (cytotoxic) increase, which, combined with the dropping level of CD4+ T-lymphocytes, produce an inversion of the CD4+/CD8+ ratio that may persist for life. However, both CD4+ and CD8+ T-lymphocyte numbers gradually decline, causing progressive dysfunction of the immune system until cats enter the third and clinical phase, during which viral replication increases and clinical disease becomes apparent [2,3,4,5]. Since FIV causes immunodeficiency syndrome by depleting CD4+ T-lymphocytes, the risk of opportunistic infections, neurological diseases, and cancer in cats increases [5,6]. In most infected cats, however, FIV itself does not cause severe clinical signs, and FIV-infected cats may live many years without any health problems [7,8].

Like HIV, FIV transmission occurs mainly through bite or vertically during pregnancy, but also via mucosal exposure and blood transfer [5,7]. Since infected cats usually develop high concentrations of FIV-specific antibodies and FIV produces a persistent infection from which cats do not recover, detection of antibodies is indicative of FIV infection. Thus, diagnosis is usually performed by using rapid ELISA or lateral flow immunoassays that detect FIV antibodies. Other assays such as immunofluorescent assay (IFA), Polymerase chain reaction (PCR) or Western blot may be used as confirmatory testing [1,4,9,10]. The purpose of this work is the development of FIVCHECK Ab ELISA, a new rapid indirect assay for the detection of FIV antibodies in feline serum/plasma samples.

## 2. Materials and Methods

### 2.1. Samples Collection

In order to both set up the new ELISA technique and assess its reliability, a total of 115 sera/plasma samples from cats with various clinical conditions were collected from 4 veterinary clinics in northern and central Italy. All samples used in this study were first tested with other assays and thus classified as positive or negative for FIV antibodies. Some of these samples were highly lipemic or hemolytic. No information was provided about breed, sex, age, clinical outcomes, disease progression, or treatments of patients with drugs, thus no sample selection was performed to contemplate the broadest possible cases.

### 2.2. ELISA Plate Preparation

Nunc PolySorp and Costar High Binding microplates (Thermo Fisher Scientific, Waltham, MA, USA) were used in the set-up of the test. All coating and blocking conditions used in the development of the new indirect ELISA test are described in standardized protocols [11,12,13] and based on internal company protocols and experience. In the preliminary phases, Nunc and Costar microplates were coated with the recombinant antigen derived from the envelope transmembrane glycoprotein gp40 of FIV at concentrations of 0.25, 0.5, 1, and 2 μg/mL in coating buffer formed by carbonate-bicarbonate (CB) buffer pH 9.6 and incubated at +2–8 °C, overnight (O/N). Successively, after a washing step with Wash buffer (Agrolabo S.p.A., Scarmagno, Italy), plates were blocked with two commercial Blocking buffers (Surmodics, Eden Prairie, MN, USA), for 1 h at room temperature (RT). The Blocking Buffer 1 has pH 7.0–7.4, contains bovine protein in phosphate-buffered saline (PBS) but not preservatives. Instead, the Blocking Buffer 2 has pH 6.5–7.5 and contains bovine protein and preservatives (0.02% methylisothiazolone and 0.02% bromonitrodioxane). The plates were then drained, left to dry, and stored at +2–8 °C.

### 2.3. FIVCHECK Ab ELISA

The new test is the result of an extensive set-up phase carried out by the Research and Development (R&D) team of Agrolabo S.p.A., considering different concentrations of antigen, conjugate, controls, and sample dilutions, to achieve the possible best test performance in terms of sensitivity, specificity, accuracy, reliability, and repeatability of results.

Nunc PolySorp microplates were prepared as previously described with 0.5 μg/mL of FIV recombinant antigen and Blocking Buffer 2. In FIVCHECK Ab ELISA, two types of positive controls (PCs) consisting of anti-histidine (His) tag antibodies labeled with horseradish peroxidase (HRP), PC1 (Abcam Limited, Cambridge, UK) and PC2 (Sigma-Aldrich, St. Louis, MO, USA), the negative control (NC), and samples, diluted 1:200 in sample diluent formed by PBS and bovine serum albumin (BSA) (Agrolabo S.p.A., Scarmagno, Italy), were distributed 100 µL in each well and incubated 10 min at RT. During this incubation, if the sample being tested contained specific anti-FIV antibodies (positive sample), they bound to the antigen attached to the wells, forming the antigen–antibody complex. After a washing step with Wash buffer in order to eliminate all the unbound material, 100 µL/well of the anti-Cat IgG conjugate antibody labeled with HRP (Abcam Limited, Cambridge, UK) was added and incubated for 10 min at RT. After a second round of washes, 100 µL of the substrate/chromogen 3,3′,5,5′-Tetramethylbenzidine (TMB) (Surmodics, Eden Prairie, MN, USA) was added and incubated for 5 min at RT in the dark, developing a colorimetric reaction (blue color was index of positive samples, no color of negative ones). Then, the reaction was stopped by adding 100 µL of Stop solution formed by sulphuric acid 0.2 M (Agrolabo S.p.A., Scarmagno, Italy), and the blue reaction turned into shades of yellow. The interpretation of results was performed by reading the optical density (OD) values at 450 nm using an ELISA microplate reader. The test was considered valid if positive control had an OD value above 0.6 and the negative control below 0.1.

The positivity or negativity of each sample was evaluated by calculating the “negative cut-off” using Equation (1) and “positive cut-off” using Equation (2) based on the OD value of the negative control:

Negative cut-off = OD negative control + 0.20(1)

Positive cut-off = OD negative control + 0.28(2)

The sample was considered negative if the OD value was lower than negative cut-off, positive if the OD value was greater than the positive cut-off, and doubtful if the OD value was between the two cut-offs.

### 2.4. FIVCHECK Ab ELISA Validation

The R&D team of Agrolabo S.p.A validated the FIVCHECK Ab ELISA by testing 115 feline sera (38 positives and 77 negatives) against the ELISA rapid test SNAP FIV/FeLV Combo (IDEXX) considered as reference. The number of samples tested was in line with that used in other bibliographic studies for validation of ELISA tests [14,15,16,17].

Briefly, in the SNAP FIV/FeLV Combo (IDEXX), 3 drops of sample were dispensed into a sample tube by using the pipette provided in the kit, then 4 drops of conjugate were added. After mixing the tube, the entire content of the sample was added carefully to the device well. The sample flowed across the result window, reaching the activation circle in a short time. When color appeared in the device activation circle, this signified that the activator had been pressed. Test results were read 10 min from the time of activation.

### 2.5. Comparative Study

A comparative study was performed by analyzing 103 sera/plasma samples (28 positives and 75 negatives) with two other rapid indirect ELISA tests widely used in veterinary practice, the INgezim FIV ELISA (Gold Standard Diagnostics, Davis, CA, USA (https://www.gsdx.us/ accessed on 9 January 2025) and VetLine FIV ELISA (NovaTec, Baltimore, MD, USA).

In the INgezim FIV ELISA (Gold Standard Diagnostics), samples diluted 1:100 and controls were added to the plate (100 μL/well) and incubated at RT for 10 min. The plate was subsequently washed and 100 μL of conjugate diluted 1:100 was added into each microwell and incubated for 10 min. Then, after another washing step, 100 μL/well of TMB substrate was added. The plate was incubated at RT for 5 min, and, finally, the reaction was stopped by adding 100 μL of stop solution to each well. Results were assessed by reading the absorbance values with a microplate reader at a wavelength of 450 nm.

In the VetLine FIV ELISA (NovaTec), a volume of 100 μL of controls and diluted samples (1:100) was added into each well, except for one well that was left empty and used for the Substrate Blank. Then, the plate was incubated for 1 h at +37 °C and, after a washing step, 100 μL of conjugate was added into all wells except for the Substrate Blank well. The plate was newly incubated for 30 min at RT. After a second round of washes, 100 μL/well of TMB substrate was added and incubated for 15 min at RT in the dark. Finally, the reaction was stopped by adding 100 μL/well of stop solution. Results were assessed by reading the absorbance values with a microplate reader at a wavelength of 450 nm.

### 2.6. Reproducibility

The ELISA’s overall precision was evaluated by performing tests at different times in the same day and on different days under similar conditions, as mentioned in the guidelines [18]. In our case, the tests were carried out twice a day (morning and afternoon, to consider any changes in ambient temperature, although the ambient temperature in the laboratory was always kept between +20–25 °C) for 14 or 15 consecutive days. Samples were repeated in duplicate for a total of 56 or 60 repetitions for each sample. All assays were conducted by two operators of the R&D team of Agrolabo S.p.A to consider operator-related variables in different analytical sessions. The test’s overall precision was defined for each sample through the evaluation of variation between wells within a single run of a plate (intra-assay) or between runs (inter-assay) by calculating the mean OD, standard deviation (SD) and minimum (Min), maximum (Max), and coefficient of variation (%CV) as the ratio between the SD and the mean OD value.

### 2.7. Stability Studies

The R&D team of Agrolabo S.p.A performed all real-time and accelerated stability studies. A single batch of ELISA plates was produced for the stability tests. Along with the plates, all reagents, such as positive/negative controls and conjugate, were prepared and then divided for the real-time stability and accelerated stability studies, to avoid the possibility of the mistaken results related to different batches’ production. In the real-time stability study, the Agrolabo’s ELISA kit was stored at +2–8 °C, in the accelerated one, it was stored at +37 °C to evaluate the aging process of the product by exposing it to elevated temperatures for a shorter time.

In the absence of specific regulations for stability testing on kits for veterinary use, the Standard Guide for Accelerated Aging of Sterile Medical Device Packages ASTM F1980-21 [19], which uses the Arrhenius equation to estimate the degradation of the medical device under accelerated conditions and represents an equivalent real-time shelf-life duration, was applied in this study. In this way, the “Accelerated Aging Time” (AAT), represented by the number of days during which the product is to be tested at the temperature in accelerated condition, was calculated through Formula (3):

(3) $$\mathrm{Accelerated}\mathrm{Aging}\mathrm{Time}\;(\mathrm{AAT})=\mathrm{Desired}\mathrm{Real}\mathrm{Time}\;(\mathrm{RT})/{Q_{10}}^{\;(T_{\mathrm{AA}}\;-\;T_{\mathrm{RT}}/10)}$$

where “RT” is the desired time at which the product is to be stored, the Accelerated Aging Temperature (T_AA_) is the test temperature in accelerated condition, the real-time temperature (T_RT_) is the temperature at which the product is to be stored, and the aging factor (Q_10_) is a measure of how quickly a material system changes when the temperature is increased by +10 °C. It is typically between 1.8 and 2.5, with a value of 2.0 being the most common value.

In our case, ELISA tests will be stored at +2–8 °C for 18 months. The time for accelerated stability study was calculated through the use of calculators found on the web [20,21] based on the Arrhenius equation reported in ASTM F1980-21, and it corresponded to 6 weeks at +37 °C. Therefore, it can be concluded that 1 week at +37 °C is equivalent to 3 months at +2–8 °C. In both real-time and accelerated stabilities, the first test was performed at the beginning of the study, at time zero (T_0_). Assays were repeated weekly for the first 6 weeks (T_1_–T_6_), testing controls and positive/negative samples with reagents stored at +2–8 °C and at +37 °C. Then, only plates at +2–8 °C were tested for real-time stability every 3 months for up to 18 months (T_7_–T_12_). In each analytical session and for each sample, the percentage remaining activity (%RA) or recovery was calculated, expressed as the ratio of the obtained OD value to that of time T_0_; the lower limit of acceptability was imposed at 70% [22]. The number of samples analyzed was in line with that used in other stability studies [15,17].

### 2.8. Cut-Off Determination

The cut-off analysis was based on data obtained in ELISA validation. The ELISA cut-off was determined by comparison of several methods. Initially, the cut-off was calculated as the mean OD value of negative samples plus 3 times the standard deviation (SD) [23,24,25], considering positive samples with OD above 10% the cut-off, negative with OD below 10% cut-off, and doubtful with OD between cut-off ±10%, as reported in other commercial ELISAs [26,27]. Moreover, the cut-off was confirmed by another common method, according to which the best cut-off would be the OD value where the difference between sensitivity and specificity is smallest [28]. According to another study [25], the optimal cut-off may be a value between the maximum OD value of negative samples (Max OD−) and the minimum OD value of positive samples (Min OD+). In addition, the Receiver Operating Characteristics (ROC) curve was used to identify the optimal cut-off value, the value that maximizes the difference between true positives and false positives. The ROC curve was constructed by joining the points obtained from the calculation of the proportion of true positives (sensitivity) and false positives (1-specificity) for all possible cut-off values. The point closest to the upper left corner of the ROC curve represented a good compromise between true and false positives and thus corresponded to the best cut-off. Closely related to the ROC curve method is the Youden’s index (J). This index was calculated for each individual cut-off by the Formula (4):

J = sensitivity + specificity − 1(4)

The parameter J takes values in the closed range [0, 1]: value 0 corresponds to a completely ineffective test, while value 1 corresponds to a perfectly effective test. The best cut-off corresponds to the maximum value among the calculated indexes.

In addition, considering the amount of true positives (a), false positives (b), false negatives (c), and true negatives (d) obtained from the FIVCHECK Ab ELISA against the reference assay, all diagnostic parameters, including the accuracy, sensitivity, specificity, positive and negative predictive values (PPV and NPV), and positive and negative likelihood ratios (LR+ and LR−) were determined at the selected cut-off value (Formulas (5)–(11)). On the sensitivity, specificity, and PPV and NPV parameters, the 95% CI was also calculated.

Diagnostic accuracy = (a + d)/(a + b + c + d)(5)

Sensitivity = a/(a + c)(6)

Specificity = d/(b + d)(7)

Positive predictive value (PPV) = a/(a + b)(8)

Negative predictive values (NPV) = d/(c + d)(9)

Positive likelihood ratio (LR+) = Sensitivity/(1 − Specificity)(10)

Negative likelihood ratio (LR−) = (1 − Sensitivity)/Specificity(11)

### 2.9. Statistical Analysis

Statistical analysis on results obtained from the comparative study was performed by ANOVA, by *t*-test considering significant a *p*-value < 0.05, and by calculating the Cohen’s d index Equation (12) to measure the strength of the relationship (Effect size). M1 and M2 are the mean OD values of the two datasets and S1 and S2 are their standard deviations.

Cohen’s d index = M1 − M2/√ (S1^2^ + S2^2^)/2 (12)

In addition, for all comparison assays, the degree of agreement was calculated by Cohen’s Kappa index Equation (13), where P_0_ is the probability of agreement observed, and P_e_ is that obtained by chance.

Cohen’s Kappa index = P_0_ − P_e_/1 − P_e_ (13)

## 3. Results

### 3.1. Plate Selection and General Assay Set-Up

PolySorp Nunc and Costar microplates (Thermo Fisher Scientific, Waltham, MA, USA) were coated with different concentrations from 0 µg/mL (non-coated plates) to 2 µg/mL of FIV recombinant antigen in CB buffer with a pH of 9.6 and blocked with Blocking Buffer 1 and Blocking Buffer 2. During ELISA preliminary assays, two samples (one positive and one negative) were diluted 1:100 in sample diluent, and the conjugate antibody anti-Cat IgG labeled with HRP was tested at different dilutions ranging from 1:2000 to 1:128,000. Non-coated plates (coating performed only with CB buffer) or coated plates tested only with the conjugate antibody diluent served as controls for aspecific binding. All diluted samples and the conjugate antibody were incubated for 10 min at RT in two successive incubations, then in the substrate TMB for 5 min at RT. Spectrophotometric readings on PolySorp Nunc and Costar microplates are shown in Table 1. All detailed OD values are reported in Supplementary Table S1.

We obtained good results on all types of plates. Considering the instrumental readings, no statistically significant differences were found between PolySorp Nunc and Costar plates coated with FIV antigen and blocked with Blocking Buffer 1 or Blocking Buffer 2, testing both the positive and negative samples. Moreover, there were no aspecific bindings with coating components and conjugate antibody diluent: in all cases, tests performed with the two samples on non-coated plates or with only conjugate antibody diluent on coated ones resulted in low OD values (OD < 0.1). In addition, considering non-coated plates, we noted a statistically significant difference (*p*-value < 0.05) between PolySorp Nunc and Costar plates blocked with Blocking Buffer 2. In detail, only by testing the positive sample, the mean OD value on PolySorp Nunc plates was statistically significantly lower than on Costar ones. The effect size was 1.595, index of great difference (Table 2). Thus, we selected the PolySorp Nunc plates blocked with Blocking Buffer 2 for future assays, since the background signal on non-coated plates was less. Moreover, on this type of plate, we selected the 0.5 µg/mL of FIV antigen as the coating concentration and the 1:8000 conjugate antibody dilution because of the better performance on both the positive and negative samples, with OD values above 1.0 not over-flowing, and lower than 0.1, respectively. In subsequent assays, the performance of the test was verified by analyzing more samples.

### 3.2. Sample Dilution Selection

To evaluate the best test performance, two sample dilutions were tested. At this purpose, samples were diluted 1:100 or 1:200 and the conjugate was used at dilution 1:8000. Samples and conjugate were incubated for 10 min at RT and TMB substrate for 5 min at RT, as in previous ELISAs. In all cases, we compared results with SNAP FIV/FeLV Combo (IDEXX) as the reference test. Initially, the cut-off was estimated in a general and simple manner as described in the literature [23,24,25] as mean OD value of negative samples plus 3 times SD, thus considering positive samples with OD above the 10% cut-off, negative with OD below the 10% cut-off and doubtful with OD values between cut-off ± 10%, as reported in other commercial ELISAs [26,27]. In the first ELISA performed on 58 feline samples (26 positives and 32 negatives) diluted 1:100, the cut-off value was set at OD 0.298; the ELISA correctly identified all positive samples. However, among negatives, six samples resulted in false positives (OD > 0.328) and one doubtful (OD values between 0.268 and 0.328). ELISA sensitivity was 100% and specificity was 81.3% (Table 3, Supplementary Table S2). Instead, in the ELISA on 113 feline samples (37 positives and 76 negatives) diluted 1:200, the cut-off value was set at OD 0.343 and the test correctly identified all samples, 37 positives and 76 negatives, and, among negatives, one sample had OD value of 0.309, thus resulting in a “doubtful” categorization (OD values between 0.309 and 0.377). ELISA sensitivity and specificity were both 100% (Table 3, Supplementary Table S3). In this context, the sample dilution 1:200 was selected because of better performances than the 1:100 one.

### 3.3. Conjugate Dilution Selection

**Figure 1:**
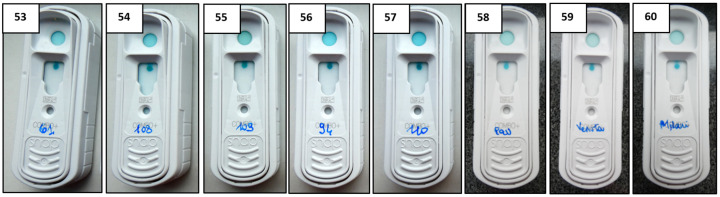
Results of SNAP FIV/FeLV Combo (IDEXX) on eight negative samples with higher OD values in ELISA. Samples: n. 53 (code: 61), n. 54 (code: 108), n. 55 (code: 109), n. 56 (code: 94), n. 57 (code: 110), n. 58 (Pau), n.59 (Venuta), n. 60 (Milani).

Despite good results obtained with conjugate antibody 1:8000, eight negative samples with SNAP FIV/FeLV Combo (IDEXX) assay had negative, but higher, OD values in our ELISA at dilution 1:200 (samples n. 28, 38, 39, 54, 57, 102, 104 and 108) (Supplementary Table S3). The results of SNAP FIV/FeLV Combo (IDEXX) on these eight samples are shown in Figure 1. To further improve the signal of negative samples, we decreased the concentration of the anti-Cat IgG HRP conjugate antibody from 1:8000 to 1:15,000. An ELISA preliminary test was performed on 60 samples diluted 1:200 (28 positives and 32 negatives, including the previously doubtful sample obtained with conjugate 1:8000). Additionally, in this case, the cut-off was estimated as described in the literature [23,24,25] as mean OD value of negative samples plus 3 times SD, thus we considered positive samples with OD above the 10% cut-off, negative with OD below the 10% cut-off, and doubtful with OD values between the cut-off ±10%, as reported previously [26,27]. The cut-off value was set at OD 0.292; FIVCHECK Ab ELISA correctly identified all positive and negative samples without doubtful results, thus improving the signal of the eight negative samples with higher OD values than had been found previously (n. 53–60) (Supplementary Table S4), in line with the results obtained with the SNAP FIV/FeLV Combo (IDEXX) assay. Since we obtained better results on negative samples even on those with higher OD values, in order to avoid losing the identification of any weakly positive samples, such as the number 27 with an OD value of 0.493 (conjugate 1:15,000) (Supplementary Table S4), we decided not to further dilute the conjugate antibody and thus maintain the 1:15,000 concentration for the validation phase and future tests.

### 3.4. Positive and Negative Controls Evaluation

Since the recombinant antigen coated on the plate contains a histidine tail and no antibodies directed to the plate-immobilized antigen are commercially available, two anti-His antibodies, PC1 and PC2, were tested as positive controls. We tested different dilutions to obtain positive, quantifiable, and non-overflow values, at least with OD values above 0.6. In fact, in an ELISA that uses TMB as substrate, a weak positive signal appears visually in a very light blue color after the addition of TMB and with an OD value of about 0.3 at 450 nm. As reported in the bibliography, a strong positive control should have an OD ≥ 1.5 [14,29]. Both positive controls worked well. As expected, since PC1 and PC2 are not developed in cat, their signals do not depend on the anti-Cat IgG HRP conjugate antibody binding. In fact, we obtained similar results by testing PC1 both with 1:8000 and 1:15,000 conjugate antibody dilutions (Table 4). Finally, we chose the dilutions 1:8000 of PC1 and 1:60,000 of PC2 because of the strong but not overflowing signals for reproducibility and stability assays. However, the performance of PC2 as positive control was better than PC1 (Table 4), in terms of the amount of reagent to be used and thus the cost, thus PC2 was ultimately chosen as positive control.

Regarding the negative control, we performed an ELISA preliminary assay by testing the sample diluent 18 times both with 1:8000 and 1:15,000 conjugate antibody dilutions. We obtained good results, since the OD values were all below 0.1 and did not exceed the OD limit of 0.3 [14,29]. In fact, the mean OD values were 0.037 and 0.035 with 1:8000 and 1:15,000 conjugate antibody, respectively. Moreover, we tested negative samples diluted 1:200 both with conjugate 1:8000 (76 samples, the same used previously in the preliminary assay) and conjugate 1:15,000 (77 samples, used then in the validation phase) to verify that they gave a signal similar to that obtained with the sample diluent. As determined by data analysis, the mean OD value of the negative control fell within the range limit of between the mean OD and the standard deviation of negative samples with both 1:8000 (0.108 ± 0.078) and 1:15,000 (0.076 ± 0.051) conjugate dilutions (Table 4).

These results made the use of commercial reagents as positive and negative controls feasible, avoiding the use of sera as potentially infectious reagents in the final kit.

### 3.5. ELISA Validation

**Figure 2:**
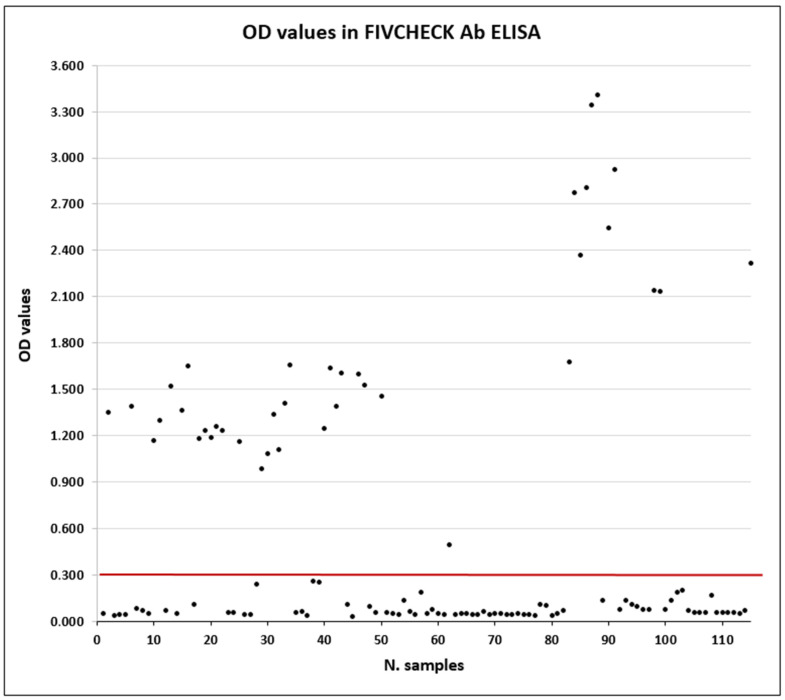
Graph showing the OD values (black dots) of the data obtained during validation. The red line indicates the OD 0.300 as the cut-off level separating negative and positive samples.

The FIVCHECK Ab ELISA was validated against the reference test SNAP FIV/FeLV Combo (IDEXX), by using 115 samples (38 positives and 77 negatives). In the course of the study, highly lipemic and hemolytic samples were also tested, but no interferences were observed. Of 115 samples tested, the FIVCHECK Ab ELISA detected 38 positives and 77 negatives (Table 5, Supplementary Table S5). The overall graph reporting the OD values of the data is shown in Figure 2. Compared to the SNAP FIV/FeLV Combo test, the FIVCHECK Ab ELISA agreed at 100%, with 100% of sensitivity (95% CI: 88.5–100%) and 100% of specificity (95% CI: 94.0–100%). The Cohen’s Kappa was 1.000, index of very good agreement. The PPV and the NPV were both 100%. Moreover, the negative likelihood ratio (LR−) was 0.000, while the positive likelihood ratio (LR+) was impossible to calculate at OD ≥ 0.270 (cut-off), but it was very high at the previous OD of 0.260 (75.00).

### 3.6. Cut-Off Evaluation

The cut-off of the FIVCHECK Ab ELISA was determined using 103 samples (37 positives and 66 negatives, sample numbers from 1 to 103, Supplementary Table S5), considering the SNAP FIV/FeLV Combo (IDEXX) the reference test.

The cut-off estimated in our previous ELISAs, as described in the literature [23,24,25,26,27] as the mean OD value of negative samples plus 3 times the SD (positive samples: OD ≥ 10% cut-off; negative samples: OD ≤ 10% cut-off; doubtful samples: OD values between cut-off ±10%), was confirmed by other methods.

**Figure 3:**
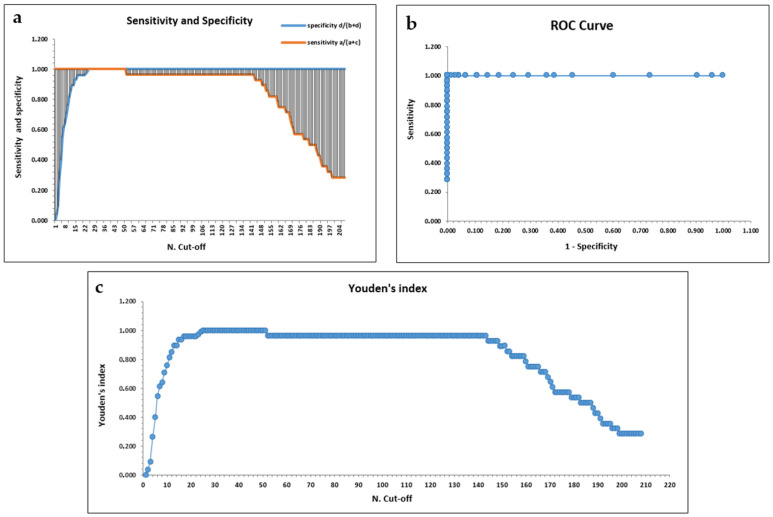
Methods for cut-off determination. Cut-off was determined by evaluating (**a**) sensibility and specificity parameters; (**b**) ROC curve; and (**c**) Youden’s index. N. Cut-off: cut-off number.

Initially, the cut-off was evaluated on sensitivity and specificity parameters as reported in the bibliography [28]: in the FIVCHECK Ab ELISA, the best cut-off corresponded to an OD value of 0.270, which was the point at which the difference between sensitivity and specificity was minimal (difference equal to zero). At the cut-off with an OD value of 0.270, sensitivity and specificity were both 100%. Moreover, the same parameters were maintained up to a value of OD 0.530 (Supplementary Table S6, Figure 3a). According to another study present in the literature [25], a correct cut-off may be a value between the maximum OD value of negative samples (Max OD−) and the minimum OD value of positive samples (Min OD+). In the FIVCHECK Ab ELISA, Max OD− was 0.261 and Min OD+ was 0.493. Based on the ROC curve, a statistical technique that identifies the optimal cut-off value, the best cut-off corresponded to an OD value of 0.270 (Figure 3b), and, according to the Youden’s index, the maximum calculated J value was 1.000 that coincided with the cut-off value with OD 0.270 (Supplementary Table S6, Figure 3c). In conclusion, since all sensitivity and specificity parameters remained the same from the cut-off with OD 0.270 up to the cut-off with OD 0.530, an OD value of 0.300 was considered as the cut-off in the final assay. Samples with OD values between 0.230 and 0.300 were considered doubtful.

### 3.7. Reproducibility Study

We performed a reproducibility study by testing the positive controls, PC1 (1:300) and PC2 (1:60,000), the NC, and one negative and two positive samples, in duplicate, twice in a day, for 14 consecutive days (56 tests for each sample) (Supplementary Table S7). The PC1 was tested only at dilution of 1:300 (reproducibility study was not performed on dilution 1:8000). In all cases, the FIVCHECK Ab ELISA was accurate, with intra- and inter-assay %CV lower than the 10% and 15–20% limits, respectively, as reported in the bibliography [22,30] (Table 6, Supplementary Table S8).

### 3.8. Comparative Analysis

The newly developed test FIVCHECK Ab ELISA was compared with two other ELISA tests, the INgezim FIV ELISA (Gold Standard Diagnostics) and VetLine FIV (NovaTec) (Supplementary Table S9).

First, we performed ANOVA statistical analysis to determine whether there were any significant differences in the OD values of positive and negative samples between the 3 types of ELISA tests. In both categories of positive and negative samples, the F values were higher than the corresponding F critical values, therefore the mean OD values of the examined three ELISAs were not equal and at least one test was significantly different (Table 7). Then, to understand which test was statistically significantly different from FIVCHECK Ab ELISA, *t*-test (considering significant a *p*-value < 0.05) and Cohen’s d index were evaluated.

#### 3.8.1. Comparative Study with INgezim FIV ELISA (Gold Standard Diagnostics)

**Figure 4:**
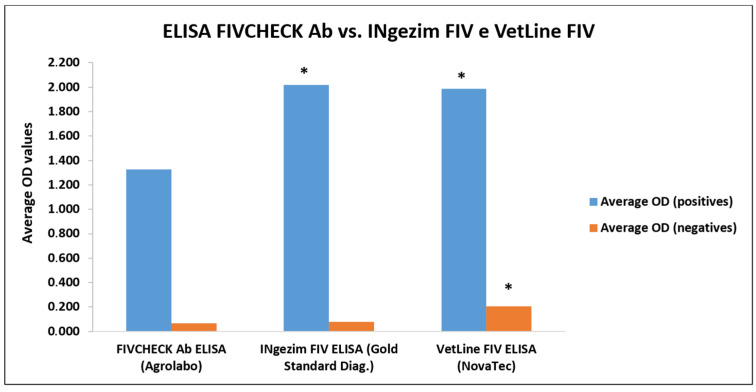
Comparative study of FIVCHECK Ab ELISA (Agrolabo), INgezim FIV ELISA (Gold Standard Diagnostics), and VetLine FIV (NovaTec). Statistical difference was assessed by *t*-test and was marked with an asterisk (*p*-value < 0.05). On positive samples, the difference between means OD was statistically significant between FIVCHECK Ab ELISA and both INgezim FIV and VetLine FIV. On negative ones, the difference between means OD was statistically significant between FIVCHECK Ab ELISA and VetLine FIV. OD: Optical density (450 nm).

All positive and negative samples were correctly identified, without false positives or false negatives, thus the new test agreed with INgezim FIV ELISA at 100% (Cohen’s Kappa: 1.000, very good agreement) (Table 8). Statistical analysis performed on mean absorbance values between the two assays found a statistically significant difference only for positive samples: the mean OD values of positive samples in the FIVCHECK Ab ELISA were statistically significantly lower than those in the INgezim FIV ELISA. The Effect size calculated by Cohen’s d gave a value of 1.956, index of great difference (Table 9; Figure 4).

#### 3.8.2. Comparative Study with VetLine FIV ELISA (NovaTec)

Of 28 positive samples, the VetLine FIV ELISA identified 26 samples, while two resulted in false negatives (positives in both FIVCHECK Ab ELISA and INgezim FIV ELISA). Moreover, of 75 negative samples, the VetLine FIV ELISA identified 69 samples, while 6 were false positives (6 negatives in both FIVCHECK Ab ELISA and FIV INgezim FIV ELISA). One negative sample in both FIVCHECK Ab ELISA and FIV INgezim FIV ELISA was doubtful in VetLine FIV ELISA (Supplementary Table S9). Thus, the FIVCHECK Ab ELISA agreed with VetLine FIV ELISA at 92.2% (Cohen’s Kappa: 0.812, very good agreement) (Table 8). Statistically significant differences were found between both positive and negative samples: in both categories, the mean OD values in the FIVCHECK Ab ELISA were statistically significantly lower than in VetLine FIV ELISA. The Effect size calculated by Cohen’s d gave values of 0.781 (negatives) and 1.662 (positives), indices of moderate and great differences, respectively (Table 9; Figure 4).

### 3.9. Stability Study

#### 3.9.1. Accelerated Stability Study

In the accelerated stability study, the PC1 (1:300 or 1:8000) and PC2 (1:60,000), the NC, and two negative and three positive samples were tested. Detailed data including spectrophotometric readings and percentage remaining activities (%RA) are shown in Supplementary Table S10. We obtained good results in all tested times with a %RA above 70% (Table 10). However, only two samples had remaining activity values slightly below 70% at T_6_ (sample 1, negative: 69.52%; sample 12, positive: 69.61%). However, at T_6_, the positive sample n.12 still had an OD value above the cut-off. Therefore, tests proved that all the reagents in the kit may be stable for 6 weeks at +37 °C and for 18 months at +2–8 °C, data confirmed with the real-time stability tests.

#### 3.9.2. Real-Time Stability Study

The real-time stability study lasted 18 months, and it was conducted with the same controls and samples already examined in the accelerated stability assays. Detailed data, including spectrophotometric readings and percentage remaining activities (%RA), are shown in Supplementary Table S11. We confirmed the results obtained with the accelerated stability: the %RA remained high, above 70% (Table 11) in all tested time points, and proved that all the reagents in the kit are stable for 18 months at the normal storage temperature +2–8 °C.

## 4. Discussion

Nunc PolySorp and Costar High Binding microplates were selected based on the Company’s in-house ELISA testing experience, and, supported by a large bibliography, used in the set-up of the test in order to choose the plate capable of providing the most accurate results. Both types of plates are commonly used in general ELISAs to immobilize antigens or antibodies. Costar High Binding microplates have hydrophobic surfaces that permit the immobilization of both medium and large biomolecules that possess ionic groups and/or hydrophobic regions. They were used both in antigens’ [31,32] and antibodies’ [33,34,35] coatings. Additionally, Nunc PolySorp plates have hydrophobic surfaces and permit the adsorption of hydrophobic molecules. They were used to immobilize both antigens [36,37] or antibodies [38] in ELISAs. However, after several tests conducted in parallel with both types of plates under analogous conditions without further variables, Nunc PolySorp microplates were chosen because of better performance results.

The FIVCHECK Ab ELISA was validated against the ELISA rapid test SNAP FIV/FeLV Combo (IDEXX), considered the reference test, by testing feline sera including positive and negative samples. The SNAP FIV/FeLV Combo (IDEXX) assay was chosen as reference method for the ELISA validation for different reasons:

1. SNAP FIV/FeLV Combo is a qualitative ELISA test, as well as FIVCHECK Ab ELISA;
2. SNAP FIV/FeLV Combo is a lateral flow ELISA that uses the FIV matrix protein p15, the capsid protein p24, and the transmembrane glycoprotein gp40 as antigens to detect FIV antibodies. Moreover, it contains monoclonal anti-FeLV p27 antibodies to detect FeLV p27 antigen [39,40,41]. However, FIVCHECK Ab ELISA uses only a recombinant antigen derived from envelope transmembrane glycoprotein gp40 because it is considered the immunodominant protein in FIV infection, instead of p15 and p24, which may be less specific for FIV and may be the result of infection with other viruses [42];
3. SNAP FIV/FeLV Combo has high sensitivity (99.2%) and high specificity (100%) for the detection of FIV antibodies [39]. In fact, for the FIV test systems, the PPV of the FIV/FeLV Combo test was very high compared to other microplate ELISA tests such as PetChek FIV antibody test (IDEXX) and Virachek FIV (Synbiotics). The PetChek FIV test contains FIV p24 and gp40 antigens, instead the Virachek FIV assay only the p24 [43]. Moreover, in a study comparing four different FIV tests using FIV-positive and FIV-negative samples, sensitivity and specificity of the SNAP FIV/FeLV Combo were 97.9% and 99.0%, respectively, compared with virus isolation as the gold standard [9,10] and PCR [44];
4. The performance of SNAP FIV/FeLV Combo assay is comparable to that of the two most currently available tests for FIV immunochromatography assay: Witness FeLV/FIV (Zoetis, Parsippany-Troy Hills, NJ, USA) and Anigen Rapid FIV/FeLV (BioNote, Hwaseong-si, Gyeonggi-do, Republic of Korea) [40].

Compared with the SNAP FIV/FeLV Combo (IDEXX), the FIVCHECK Ab ELISA correctly identified FIV positive and negative samples with very high sensitivity (100%, 95% CI: 88.5–100%) and high specificity (100%, 95% CI: 94.0–100%).

Interpretation of the results was performed instrumentally. From data analysis, the FIVCHECK Ab ELISA cut-off was set at OD 0.300, and, in the final kit, the user will determine the positivity or negativity of the samples by calculating the “negative cut-off” and “positive cut-off” formulas based on the OD value of the negative control. Thus, the sample will be considered negative if the OD value is lower than negative cut-off, positive if the OD value is greater than the positive cut-off, and doubtful if the OD value is between the two cut-offs. In this latter instance, it is recommended to repeat the analysis or take another sample a couple of weeks later.

No interference was observed on highly lipemic or hemolytic samples collected in different types of anticoagulants in the FIVCHECK Ab ELISA. However, the presence of ELISA-interfering substances, including drugs or natural compounds, in the blood of cats with profound inflammation or immune activation should be explored. In this regard, we did not perform extensive studies to verify cross-reactivity reactions, partly due to a lack of representative samples, which are difficult to find.

We did not report false positive or false negative results. In antibody assays using indirect ELISA, false positive and negative reactions can occur due to various reasons unrelated to the antigens coated to the plate. In fact, during sample preparation, errors such as sample collection, storage, handling, and processing contamination can interfere with the accuracy of the test results. Generally, ELISA false positive reactions may be caused by nonspecific reactivity and background noise resulting from the binding of antigens or immunoglobulins in the sample specimens to the solid phase. In addition, cross-reactivity reactions may occur when ELISA test detects antibodies or antigens that are similar to, but not identical to, the target antigen or antibody. However, this reaction is dependent on each sample and can vary significantly, sometimes even surpassing the true antibody–antigen reaction. Although we tested a large number of samples, this step is sample-dependent and difficult to predict.

FIV false positive results may occur in uninfected FIV-vaccinated cats. For this reason, the ELISA antibody assays for the detection of FIV antibodies alone cannot be used to distinguish FIV-vaccinated/FIV-uninfected cats from FIV-infected cats. In addition, maternally derived anti-FIV antibodies may be present in kittens for up to 3 months, and it may be a further two months before infected kittens seroconvert [5]. Moreover, the exposure to off-target pathogens may elicit antibodies that may non-specifically bind to antigens used in ELISAs. In a study reported in the literature, domestic cats with multiple pathogen exposures and ongoing infections resulted in antibodies that cross-reacted non-specifically with the FIV Gag antigen. In that case, 3 of 12 FIV infected cats had higher seroreactivity to the Gag antigen of the retrovirus feline foamy virus (FFV) [45]. Cross-reaction studies in cats were observed by testing other feline lentiviruses compared with FIV, such as puma lentivirus PLV-A and PLV-B or lion lentivirus FIV_Ple_, which typically resulted in lower levels of plasma viremia than host-adapted FIV strains [46]. In another study, PLV and FIV co-infected cats had delayed FIV proviral or RNA load detection in blood and had accelerated anti-FIV capsid antibody development when compared to cats with single PLV or single FIV infection [47]. However, FIV positive cats tested with the FIV ELISA based on FIV Gag and gp40 antigens were negative in PLV ELISA and PLV Western blot, suggesting the absence of cross-reactivity [48].

Regarding false negative results, they may occur early in the disease before there is a sufficient antibody response or late in the disease when the cat is severely immunosuppressed [49]. Considering the animal’s clinical signs, false positive and false negative FIV tests should be retested after a few weeks or confirmed by another assay, such as IFA, PCR, or Western blot.

No information was provided about breed, sex, age, clinical outcomes, disease progression, or treatments of patients with drugs, thus no sample selection was performed in order to contemplate the broadest possible cases. In this way, we also simulated the case of clinicians who might analyze specimens from patients with unknown diagnoses or general conditions. Therefore, our ELISA resulted independently from physiological and pathological conditions of the patient animal. However, further clinical experience and additional patient outcome data will help to reveal more about the specific mechanisms underlying cross-reactive antibody production. In this context, additional future work could be directed to exploring factors that may impact test results.

Although a real-world study is lacking, the company has not received any reports of test malfunction by end-users since the product was put on the market (two years). The test’s correct functioning is evidenced by a recent study reported in the literature in which feline samples were analyzed for FIV antibodies first by lateral flow assay, then confirmed by using the FIVCHECK Ab ELISA [50].

The new ELISA is an accurate assay with intra- and inter-assay %CV lower than 20%, defined as the limit of acceptable %CV for precision and accuracy in the general guidelines [22,30]. Regarding ELISA kit controls, we evaluated commercial reagents for reasons related to future production needs and safety for the end-user. In fact, the availability of samples, especially positives to be used as positive controls, is limited, and the amount to be collected and put in the final kit during test production would be too much. In addition, the use of serum/plasma as control (especially positive) might result in variability and lack of homogeneity between batches due to the nature of the sample (strong positive or weak positive). Moreover, to ensure operator safety, it is good practice to avoid the use of samples that are potentially infected, so we included in the kit only commercial reagents certified as non-infectious.

Histidine tags are characterized by low immunogenicity, small size, and are indispensable tools in many research laboratories, as typically they are the most widely used affinity tags for purifying recombinant proteins under both native and denaturing conditions [51]. In our case, since the FIV antigen coated on the plate contains a histidine tail and no antibodies directed to the plate-immobilized antigen are commercially available, two anti-His antibodies were tested as positive controls. The use of antibodies that bind to His-tagged proteins has been reported in the literature [52,53,54,55]. In order to obtain a strong positive control, a minimum OD value of 0.6 was chosen for test validity as reported in bibliography [14,29] and in ELISAs [56,57,58]. Although both reagents tested gave good results, we demonstrated that PC2 might be an excellent positive control in FIVCHECK Ab ELISA because of the best performances.

Instead, the negative control should not exceed an OD of 0.3 [14,29] and, in our developed ELISA, sample diluent used as the negative control always gave OD values below 0.1. It is known that PBS-based solutions are used as negative controls both in ELISA tests developed for research purposes and in commercially available assays [59,60,61,62,63]. Here, we demonstrated the feasibility of using sample diluent as the negative control. In fact, the mean OD value of the negative control fell within the range limit obtained by analyzing negative samples with both 1:8000 and 1:15,000 conjugate dilutions.

A comparative study was performed by analyzing samples with two other rapid indirect ELISA tests widely used in the literature, the INgezim FIV ELISA (Gold Standard Diagnostics) [64,65] and VetLine FIV ELISA (NovaTec) [66,67,68]. Excellent agreement (100%, Cohen’s Kappa of 1.000) resulted from the comparative study, with INgezim FIV ELISA as all positive and negative samples were correctly identified, without false positives or false negatives. Instead, very good agreement was found with VetLine FIV ELISA at 92.2% (Cohen’s Kappa: 0.812), even if two samples resulted in false negatives and six false positives. However, we found a statistically significant difference between both positive and negative samples among the three compared ELISA tests. In particular, in FIVCHECK Ab ELISA, positive samples had statistically significant lower OD values compared to INgezim FIV and VetLine FIV, with great differences (Effect sizes of 1.956 and 1.662, respectively). In addition, negative samples had significantly lower OD values in FIVCHECK Ab ELISA compared with the VetLine FIV (Effect size of 0.781, moderate difference). In this context, in the FIVCHECK Ab ELISA, results may appear clearer and could be interpreted in a better way.

The ELISA kit must be refrigerated at +2–8 °C to ensure its stability from production to use. However, accelerated stability is normally performed at +37 °C to accelerate the chemical or physical reactions of the biological reagents [69,70], and it is the most suitable method for simulate product degradation and thus to determine its expiration quickly, without waiting a long period for degradation to actually occur. In fact, many unexpected factors, such as cold chain breakage, may arise during transportation or storage, and they may negatively impact the quality and performance of the product.

The climatic zones where the products are sold determine the storage conditions that should be used. Depending on country-specific regulations, stability tests may need to be conducted at defined temperatures reflecting the respective climate zone of the country in which it is intended to market. If the product is stable under the storage conditions of the hotter climatic zone, then it is automatically suitable for use in colder zones [71,72]. The temperature of +37 °C simulates that of hotter climate zones; thus, at the end of stability testing, the product can be marketed in all climate zones, including III (hot and dry climate) and IV (hot and humid/very humid climate).

In contrast to pharmaceutical products or veterinary drugs for which there are present guidelines for stability testing [73,74], the prediction of the shelf-life related to veterinary diagnostic kits (ELISA or immunochromatographic assays) does not refer to any specific legislation or regulations. In the absence of standards regarding stability tests related to veterinary diagnostic kits, reference was made to existing methods used for medical devices. In particular, we utilized the Standard Guide for Accelerated Aging of Sterile Medical Device Packages ASTM F1980-21 [19] that uses the Arrhenius equation to estimate the degradation of a medical device under accelerated conditions and represents an equivalent real-time shelf-life duration. According to this guideline, stability studies conducted on the FIVCHECK Ab ELISA demonstrated that the kit is stable for 18 months at +2–8 °C and for 6 weeks at +37 °C. This makes the product suitable for use even in hotter climate zones and in conditions of possible cold chain breakage for up to 6 weeks.

## 5. Conclusions

This study demonstrates that the FIVCHECK Ab ELISA is an accurate test characterized by high sensitivity and specificity. The new FIVCHECK Ab ELISA provided correct and reliable results very quickly (25 min) without the need for thermostats. The interpretation of results is performed instrumentally by reading the OD values at 450 nm using an ELISA microplate reader. The new test is also suitable for the analysis of highly lipemic or hemolytic samples, as no interferences were observed. The new ELISA contains safe reagents for the end-user and has a long-term storage up to 18 months at +2–8 °C. All these features make this test optimal for use in veterinary practice.

## Figures and Tables

**Table 1:** ELISA preliminary assays on PolySorp Nunc and Costar microplates. Test ELISA on PolySorp Nunc and Costar High Binding microplates blocked with Blocking Buffer 1 and Buffer 2. Coating: 0, 0.25, 0.5, 1 and 2 µg/mL FIV recombinant antigen. Samples: 1 positive and 1 negative (dilution 1:100). Conjugate was tested from 1:2000 to 1:128,000.

Samples	Conjugate	PolySorp Nunc Plate										Costar High Binding									
		Blocking Buffer 1					Blocking Buffer 2					Blocking Buffer 1					Blocking Buffer 2				
		2	1	0.5	0.25	0	2	1	0.5	0.25	0	2	1	0.5	0.25	0	2	1	0.5	0.25	0
Positive	1:2000	2.648	2.674	2.609	2.234	0.056	2.624	2.606	2.597	2.529	0.043	2.683	2.668	2.642	2.552	0.088	2.654	2.674	2.643	2.572	0.077
	1:4000	2.637	2.620	2.428	1.480	0.040	2.601	2.585	2.475	2.083	0.036	2.673	2.621	2.564	2.210	0.060	2.651	2.659	2.586	2.332	0.054
	1:8000	2.474	2.342	1.764	0.838	0.038	2.398	2.305	1.928	1.316	0.035	2.555	2.455	2.151	1.504	0.049	2.544	2.485	2.243	1.664	0.045
	1:16,000	1.941	1.647	1.065	0.475	0.036	1.800	1.628	1.191	0.717	0.030	2.096	1.903	1.448	0.866	0.037	2.112	1.915	1.517	0.987	0.040
	1:32,000	1.159	0.928	0.580	0.249	0.037	1.059	0.919	0.681	0.404	0.030	1.327	1.129	0.818	0.441	0.035	1.301	1.146	0.864	0.528	0.036
	1:64,000	0.652	0.528	0.323	0.148	0.032	0.635	0.569	0.406	0.238	0.030	0.775	0.662	0.466	0.256	0.045	0.743	0.648	0.487	0.299	0.046
	1:128,000	0.339	0.273	0.175	0.090	0.035	0.345	0.299	0.215	0.139	0.030	0.404	0.339	0.258	0.147	0.040	0.402	0.352	0.269	0.175	0.043
	0	0.032	0.033	0.034	0.028	0.032	0.048	0.029	0.030	0.028	0.027	0.048	0.035	0.035	0.032	0.035	0.030	0.032	0.031	0.032	0.044
Negative	1:2000	0.233	0.133	0.087	0.068	0.070	0.173	0.114	0.066	0.051	0.041	0.260	0.137	0.096	0.082	0.162	0.237	0.117	0.077	0.061	0.057
	1:4000	0.127	0.083	0.055	0.047	0.044	0.102	0.075	0.055	0.041	0.039	0.146	0.090	0.071	0.060	0.060	0.138	0.070	0.052	0.047	0.045
	1:8000	0.083	0.056	0.041	0.036	0.036	0.069	0.052	0.039	0.034	0.032	0.098	0.061	0.053	0.050	0.044	0.087	0.053	0.043	0.040	0.037
	1:16,000	0.052	0.044	0.034	0.032	0.035	0.047	0.040	0.033	0.034	0.036	0.076	0.046	0.043	0.041	0.040	0.077	0.042	0.038	0.040	0.037
	1:32,000	0.043	0.034	0.035	0.029	0.031	0.039	0.035	0.034	0.034	0.030	0.051	0.066	0.039	0.039	0.037	0.048	0.042	0.035	0.042	0.038
	0	0.028	0.028	0.029	0.028	0.029	0.031	0.030	0.033	0.031	0.029	0.037	0.035	0.035	0.037	0.036	0.035	0.036	0.034	0.034	0.036

**Table 2:** ELISA preliminary assays performed on PolySorp Nunc and Costar microplates blocked with Blocking Buffer 1 and Buffer 2. Coating: 0, 0.25, 0.5, 1 and 2 µg/mL FIV recombinant antigen. Samples: 1 positive and 1 negative (dilution 1:100). Statistical analysis was performed by *t*-test considering significant a *p*-value < 0.05 and by calculation of the Cohen’s d index (Effect size). Statistically significant data are marked with an asterisk. Cohen’s d (Effect size): d = 0.2 small effect, d = 0.5 moderate effect, d = 0.8 great effect.

	Antigen (µg/mL)	Plate	Positive Sample			Negative Sample		
			Mean OD	*p*-Value	Effect Size	Mean OD	*p*-Value	Effect Size
Blocking Buffer 1	2	Costar	1.788	0.856	-	0.126	0.723	-
		Nunc	1.693			0.108		
	1	Costar	1.682	0.840	-	0.080	0.686	-
		Nunc	1.573			0.070		
	0.5	Costar	1.478	0.713	-	0.060	0.510	-
		Nunc	1.278			0.050		
	0.25	Costar	1.139	0.473	-	0.054	0.290	-
		Nunc	0.788			0.042		
	0	Costar	0.049	0.168	-	0.063	0.329	-
		Nunc	0.038			0.041		
Blocking Buffer 2	2	Costar	1.772	0.796	-	0.117	0.468	-
		Nunc	1.637			0.086		
	1	Costar	1.697	0.796	-	0.065	0.939	-
		Nunc	1.559			0.063		
	0.5	Costar	1.516	0.820	-	0.049	0.731	-
		Nunc	1.637			0.045		
	0.25	Costar	1.222	0.758	-	0.046	0.202	-
		Nunc	1.061			0.039		
	0	Costar	0.048	0.011 *	1.595	0.042	0.096	-
		Nunc	0.033			0.035		

**Table 3:** Results of FIVCHECK Ab ELISA obtained from sample dilution 1:100 or 1:200. ELISA performances were compared against the reference test SNAP FIV/FeLV Combo (IDEXX). Sample dilution 1:100 resulted in ELISA sensitivity of 100% and specificity of 81.3%. Sample dilution 1:200 resulted in ELISA sensitivity of 100% and specificity of 100%. (a) true positives; (b) false positives; (c) false negatives; (d) true negatives.

Sample Dilution	FIVCHECK Ab ELISA (Agrolabo)	SNAP FIV/FeLV Combo (IDEXX)		
		Positive	Negative	Total
1:100	Positive	26 (a)	6 (b)	32
	Negative	0 (c)	26 (d)	26
	Total	26	32	58
1:200	Positive	37 (a)	0 (b)	37
	Negative	0 (c)	76 (d)	76
	Total	37	76	113

**Table 4:** Evaluation of positive and negative controls in FIVCHECK Ab ELISA. Conjugate antibody was tested at dilutions 1:8000 or 1:15,000. Results are expressed as mean OD ± standard deviation. PC1 and PC2: positive controls anti-His HRP antibody; NC: negative control (sample diluent). Negative samples were tested diluted 1:200 with conjugate antibody 1:8000 or 1:15,000. n: number of tests; n.d.: not determined.

Samples	Dilution	Conjugate	
		1:8000	1:15,000
PC1	1:2000	2.642 ± 0.008 (n = 2)	n.d.
	1:4000	2.319 ± 0.004 (n = 2)	n.d.
	1:8000	1.987 ± 0.009 (n = 2)	2.006 ± 0.036 (n = 3)
	1:10,000	1.467 ± 0.015 (n = 6)	1.479 ± 0.062 (n = 2)
	1:12,000	1.248 ± 0.041 (n = 4)	1.293 ± 0.010 (n = 2)
	1:16,000	0.931 ± 0.134 (n = 4)	0.811 ± 0.001 (n = 2)
PC2	1:2000	n.d.	>4.000 (n = 3)
	1:4000	n.d.	>4.000 (n = 4)
	1:5000	n.d.	>4.000 (n = 3)
	1:8000	n.d.	>4.000 (n = 4)
	1:10,000	n.d.	>4.000 (n = 3)
	1:12,000	n.d.	>4.000 (n = 4)
	1:16,000	n.d.	>4.000 (n = 4)
	1:20,000	n.d.	>4.000 (n = 3)
	1:30,000	n.d.	3.618 ± 0.060 (n = 3)
	1:40,000	n.d.	3.237 ± 0.071 (n = 3)
	1:50,000	n.d.	2.762 ± 0.049 (n = 3)
	1:60,000	n.d.	2.569 ± 0.080 (n = 3)
NC	-	0.037 ± 0.002 (n = 18)	0.035 ± 0.003 (n = 18)
Negative samples	1:200	0.108 ± 0.078 (n = 76)	0.076 ± 0.051 (n = 77)

**Table 5:** Validation results of FIVCHECK Ab ELISA (Agrolabo) against the reference test SNAP FIV/FeLV Combo (IDEXX). Total number of samples analyzed: 115. (a) true positives; (b) false positives; (c) false negatives; (d) true negatives. Proportion of agreement (a + d/a + b + c + d): 100%; Cohen’s Kappa: 1.000; Sensitivity: 100%; Specificity: 100%.

FIVCHECK Ab ELISA (Agrolabo)	SNAP FIV/FeLV Combo (IDEXX)		
	Positive	Negative	Total
Positive	38 (a)	0 (b)	38
Negative	0 (c)	77 (d)	77
Total	38	77	115

**Table 6:** Summary of the reproducibility study. For each sample, the overall number of tests (N), the mean OD, standard deviation (SD), %CV, the median, variance, mode, the minimum (Min) and maximum (Max) OD values, and %CV for intra- and inter-assay were reported. PC1: positive control anti-His HRP antibody (1:300); PC2: a positive control anti-His HRP antibody (1:60,000); NC: negative control; sample 121 (negative), sample 3 (positive), sample 7 (positive).

Sample	N	Mean OD	SD	%CV	Min OD	Max OD	Median	Variance	Mode OD	Intra-Assay %CV		Inter-Assay %CV	
										Min	Max	Min	Max
PC1	56	2.700	0.039	1.443	2.621	2.793	2.697	0.001490	2.706	0.052	2.246	0.175	1.977
PC2	56	3.165	0.036	1.140	3.112	3.258	3.162	0.001280	3.169	0.044	1.836	0.044	1.019
NC	56	0.035	0.002	4.579	0.032	0.041	0.035	0.000003	0.033	0.000	9.959	0.000	8.081
Sample 121 (−)	56	0.039	0.003	8.647	0.033	0.057	0.039	0.000016	0.038	0.000	10.607	0.760	8.772
Sample 3 (+)	56	1.497	0.030	2.014	1.427	1.530	1.510	0.000893	1.520	0.000	3.235	0.070	3.145
Sample 7 (+)	56	1.746	0.031	1.799	1.659	1.808	1.754	0.000969	1.742	0.000	2.760	0.085	2.698

**Table 7:** Analysis of variance by ANOVA on FIVCHECK Ab, INgezim FIV and VetLine FIV ELISA. SS: sum-of-squares terms; df: degrees of freedom; MS: mean squares (variances); F: F value resulted from ANOVA; F crit: critical value F; n: number of samples.

Samples	Origin of Variation	SS	df	MS	F	*p*-Value	F Crit
Positives (n = 28)	Between groups	8.495	2	4.247	25.611	2.4 × 10^−9^	3.109
	In groups	13.434	81	0.166			
	Total	21.929	83				
Negatives (n = 75)	Between groups	0.871	2	0.435	19.919	1.1 × 10^−8^	3.036
	In groups	4.853	222	0.022			
	Total	5.724	224				

**Table 8:** Comparison of FIVCHECK Ab ELISA (Agrolabo) with INgezim FIV ELISA (GSD) and VetLine FIV (NovaTec). Total number of samples analyzed: 103; (a) true positives; (b) false positives; (c) false negatives; (d) true negatives. Comparison results of FIVCHECK Ab ELISA and INgezim FIV ELISA: proportion of agreement (a + d/a + b + c + d) of 100% and Cohen’s Kappa of 1.000. Comparison results of FIVCHECK Ab ELISA and VetLine FIV: proportion of agreement (a + d/a + b + c + d): 92.2%; Cohen’s Kappa: 0.812. GSD: Gold Standard Diagnostics.

Assay		FIVCHECK Ab ELISA (Agrolabo)		
		Positive	Negative	Total
INgezim FIV ELISA (GSD)	Positive	28 (a)	0 (b)	28
	Negative	0 (c)	75 (d)	75
	Total	28	75	103
VetLine FIV (NovaTec)	Positive	26 (a)	6 (b)	32
	Negative	2 (c)	69 (d)	71
	Total	28	75	103

**Table 9:** Comparative study and statistical analysis. Statistical difference was assessed by *t*-test (*p*-value < 0.05) and Cohen’s d on FIVCHECK Ab ELISA (Agrolabo), INgezim FIV (Gold Standard Diagnostics, GSD) and VetLine FIV (NovaTec). Cohen’s d (Effect size): d = 0.2 small effect, d = 0.5 moderate effect, d = 0.8 great effect.

	Positive Samples			Negative Samples		
	ELISA (Agrolabo)	INgezim FIV	VetLine FIV	ELISA (Agrolabo)	INgezim FIV	VetLine FIV
N. Tests	28/28	28/28	32/28	75/75	75/75	71/75
Mean OD	1.329	2.020	1.985	0.066	0.080	0.204
*p*-value (<0.05)	-	3.9 × 10^−9^	2.3 × 10^−7^	-	0.081	7.7 × 10^−6^
Cohen’s d	-	1.956	1.662	-	-	0.781

**Table 10:** Accelerated stability study. Analysis times: T_0_–T_6_. OD: mean of spectrophotometric readings; %RA: percentage remaining activities; PC1: positive control anti-His HRP antibody 1:300 or 1:8000; PC2: positive control anti-His HRP antibody 1:60,000; NC: negative control; negative samples: 1, 6; positive samples: 11, 12, 3.

Samples	T_0_		T_1_		T_2_		T_3_		T_4_		T_5_		T_6_	
	OD	%RA	OD	%RA	OD	%RA	OD	%RA	OD	%RA	OD	%RA	OD	%RA
PC1 1:300	2.682	100	2.679	99.91	2.654	98.97	2.648	98.75	2.631	98.10	2.611	97.35	2.586	96.42
PC1 1:8000	2.241	100	2.030	90.56	1.981	88.38	1.881	83.91	1.843	82.22	1.849	82.49	1.717	76.59
PC2 1:60,000	3.138	100	3.128	99.70	3.039	96.86	2.939	93.66	2.823	89.96	2.461	78.44	2.246	71.59
NC	0.034	100	0.032	94.03	0.032	94.03	0.031	92.54	0.029	86.57	0.030	89.55	0.028	83.58
Sample 1 (−)	0.053	100	0.049	92.38	0.047	89.52	0.046	86.67	0.042	79.05	0.040	75.24	0.037	69.52
Sample 6 (−)	0.041	100	0.039	95.12	0.038	91.46	0.034	82.93	0.033	80.49	0.037	90.24	0.031	75.61
Sample 11 (+)	1.708	100	1.715	100.41	1.538	90.02	1.517	88.82	1.295	75.82	1.242	72.69	1.210	70.84
Sample 12 (+)	1.478	100	1.492	100.98	1.354	91.64	1.185	80.20	1.156	78.24	1.083	73.30	1.029	69.61
Sample 3 (+)	1.369	100	1.375	100.47	1.370	100.11	1.234	90.14	1.180	86.19	1.022	74.68	0.999	73.00

**Table 11:** Real-time stability study. Analysis times: T_0_–T_12_. OD: mean of spectrophotometric readings; %RA: percentage remaining activities; PC1: positive control anti-His HRP antibody 1:300 or 1:8000; PC2: positive control anti-His HRP antibody 1:60,000; NC: negative control; negative samples: 1, 6; positive samples: 11, 12, 3.

Samples	T_0_		T_1_		T_2_		T_3_		T_4_		T_5_		T_6_		T_7_		T_8_		T_9_		T_10_		T_11_		T_12_	
	OD	%RA	OD	%RA	OD	%RA	OD	%RA	OD	%RA	OD	%RA	OD	%RA	OD	%RA	OD	%RA	OD	%RA	OD	%RA	OD	%RA	OD	%RA
PC1 1:300	2.682	100	2.690	100.30	2.636	98.30	2.634	98.21	2.629	98.04	2.631	98.12	2.650	98.83	2.640	98.45	2.664	99.33	2.623	97.82	2.632	98.15	2.498	93.15	2.387	89.00
PC1 1:8000	2.241	100	2.243	100.07	2.245	100.16	2.243	100.09	2.242	100.02	2.246	100.20	2.241	100.00	2.363	105.42	2.240	99.93	1.903	84.90	1.924	85.83	1.881	83.91	1.637	73.05
PC2 1:60,000	3.138	100	3.132	99.81	3.123	99.54	3.124	99.55	3.166	100.89	2.923	93.15	2.867	91.38	2.870	91.46	2.710	86.36	2.697	85.94	2.602	82.93	2.467	78.63	2.364	75.35
NC	0.034	100	0.034	100	0.029	86.57	0.031	91.04	0.030	88.06	0.029	86.57	0.030	88.06	0.028	83.58	0.030	88.06	0.029	86.57	0.030	88.06	0.030	89.20	0.029	88.00
Sample 1	0.053	100	0.048	90.48	0.045	85.71	0.045	84.76	0.046	86.67	0.043	81.90	0.046	87.62	0.045	84.76	0.043	80.95	0.040	76.19	0.045	85.71	0.042	80.31	0.041	77.47
Sample 6	0.041	100	0.039	93.90	0.039	93.90	0.039	93.90	0.035	85.37	0.034	81.71	0.036	86.59	0.038	91.46	0.043	104.88	0.043	103.66	0.045	108.54	0.038	93.25	0.036	87.49
Sample 11	2.708	100	2.866	105.84	2.866	105.83	2.864	105.76	2.845	105.06	2.861	105.65	2.863	105.71	2.865	105.78	2.531	93.45	2.169	80.10	2.241	82.74	2.199	81.20	2.045	75.51
Sample 12	2.478	100	2.684	108.34	2.635	106.36	2.632	106.24	2.634	106.30	2.646	106.78	2.648	106.86	2.642	106.64	2.512	101.37	2.047	82.62	1.849	74.63	1.841	74.30	1.734	70.00
Sample 3	2.369	100	2.476	104.54	2.469	104.22	2.465	104.07	2.470	104.26	2.475	104.50	2.477	104.58	2.476	104.52	2.541	107.28	2.082	87.88	1.867	78.83	1.806	76.25	1.730	73.02

## Data Availability

Data are available in this published article and as Supplementary Information. Datasets generated during the current study are also available from the corresponding author on reasonable request.

## Supplementary Materials

The following supporting information can be downloaded at: www.mdpi.com/article/10.3390/vetsci12020089/s1, Supplementary Table S1. ELISA preliminary assays. Supplementary Table S2. Results of FIVCHECK Ab ELISA (sample dilution 1:100) against the SNAP FIV/FeLV Combo (IDEXX). Supplementary Table S3. Results of FIVCHECK Ab ELISA (sample dilution 1:200) against the SNAP FIV/FeLV Combo (IDEXX). Supplementary Table S4. Conjugate antibody selection in FIVCHECK Ab ELISA. Supplementary Table S5. Validation results of FIVCHECK Ab ELISA against the reference test SNAP FIV/FeLV Combo (IDEXX). Supplementary Table S6. Cut-off determination. Supplementary Table S7. Reproducibility study. Supplementary Table S8. Intra- and inter-assay coefficient of variation of FIVCHECK Ab ELISA. Supplementary Table S9. Comparison results of FIVCHECK Ab ELISA, INgezim FIV and VetLine. Supplementary Table S10. Accelerated stability study. Supplementary Table S11. Real-time stability study.
