# Supplementary material for: A New Rapid Indirect ELISA Test for Serological Diagnosis of Feline Immunodeficiency
Source: Vet Sci. 2025 Jan 23;12(2):89. doi: 10.3390/vetsci12020089 (PMC11860318; doi:10.3390/vetsci12020089)
Supplement: Supplementary file 1 [file vetsci-12-00089-s001.zip › vetsci-3394382-supplementary.pdf]

Supplementary Table S1. ELISA preliminary assays.

Test ELISA on Costar High Binding or PolySorp Nunc microplates blocked with Blocking Buffer 1 or in Blocking Buffer 2. Coating: 0, 0.25, 0.5, 1 and 2 μg/mL FIV recombinant antigen. Samples: 1 positive and 1 negative (dilution 1:100). Conjugate: anti-Cat IgG HRP antibody diluted 1:2,000 – 1:128,000. Incubation times: 10 minutes at RT (samples), 10 minutes at RT (conjugate antibody) and 5 minutes, RT (TMB). n.d.: not determined.

**Microplate**	**Blocking Buffer**	**Conjugate**	**Positive sample**					**Negative sample**				
			**2**	**1**	**0.5**	**0.25**	**0**	**2**	**1**	**0.5**	**0.25**	**0**
Costar High Binding	Blocking Buffer 1	1:2,000	2.683	2.668	2.642	2.552	0.088	0.260	0.137	0.096	0.082	0.162
		1:4,000	2.673	2.621	2.564	2.210	0.060	0.146	0.090	0.071	0.060	0.060
		1:8,000	2.555	2.455	2.151	1.504	0.049	0.098	0.061	0.053	0.050	0.044
		1:16,000	2.096	1.903	1.448	0.866	0.037	0.076	0.046	0.043	0.041	0.040
		1:32,000	1.327	1.129	0.818	0.441	0.035	0.051	0.066	0.039	0.039	0.037
		1:64,000	0.775	0.662	0.466	0.256	0.045	n.d.	n.d.	n.d.	n.d.	n.d.
		1:128,000	0.404	0.339	0.258	0.147	0.040	n.d.	n.d.	n.d.	n.d.	n.d.
		0	0.048	0.035	0.035	0.032	0.035	0.037	0.035	0.035	0.037	0.036
	Blocking Buffer 2	1:2,000	2.654	2.674	2.643	2.572	0.077	0.237	0.117	0.077	0.061	0.057
		1:4,000	2.651	2.659	2.586	2.332	0.054	0.138	0.070	0.052	0.047	0.045
		1:8,000	2.544	2.485	2.243	1.664	0.045	0.087	0.053	0.043	0.040	0.037
		1:16,000	2.112	1.915	1.517	0.987	0.040	0.077	0.042	0.038	0.040	0.037
		1:32,000	1.301	1.146	0.864	0.528	0.036	0.048	0.042	0.035	0.042	0.038
		1:64,000	0.743	0.648	0.487	0.299	0.046	n.d.	n.d.	n.d.	n.d.	n.d.
		1:128,000	0.402	0.352	0.269	0.175	0.043	n.d.	n.d.	n.d.	n.d.	n.d.
		0	0.030	0.032	0.031	0.032	0.044	0.035	0.036	0.034	0.034	0.036
PolySorp Nunc	Blocking Buffer 1	1:2,000	2.648	2.674	2.609	2.234	0.056	0.233	0.133	0.087	0.068	0.070
		1:4,000	2.637	2.620	2.428	1.480	0.040	0.127	0.083	0.055	0.047	0.044
		1:8,000	2.474	2.342	1.764	0.838	0.038	0.083	0.056	0.041	0.036	0.036
		1:16,000	1.941	1.647	1.065	0.475	0.036	0.052	0.044	0.034	0.032	0.035
		1:32,000	1.159	0.928	0.580	0.249	0.037	0.043	0.034	0.035	0.029	0.031
		1:64,000	0.652	0.528	0.323	0.148	0.032	n.d.	n.d.	n.d.	n.d.	n.d.
		1:128,000	0.339	0.273	0.175	0.090	0.035	n.d.	n.d.	n.d.	n.d.	n.d.
		0	0.032	0.033	0.034	0.028	0.032	0.028	0.028	0.029	0.028	0.029
	Blocking Buffer 2	1:2,000	2.624	2.606	2.597	2.529	0.043	0.173	0.114	0.066	0.051	0.041
		1:4,000	2.601	2.585	2.475	2.083	0.036	0.102	0.075	0.055	0.041	0.039
		1:8,000	2.398	2.305	1.928	1.316	0.035	0.069	0.052	0.039	0.034	0.032
		1:16,000	1.800	1.628	1.191	0.717	0.030	0.047	0.040	0.033	0.034	0.036
		1:32,000	1.059	0.919	0.681	0.404	0.030	0.039	0.035	0.034	0.034	0.030
		1:64,000	0.635	0.569	0.406	0.238	0.030	n.d.	n.d.	n.d.	n.d.	n.d.
		1:128,000	0.345	0.299	0.215	0.139	0.030	n.d.	n.d.	n.d.	n.d.	n.d.
		0	0.048	0.029	0.030	0.028	0.027	0.031	0.030	0.033	0.031	0.029

Supplementary Table S2. Results of FIVCHECK Ab ELISA (sample dilution 1:100) against the SNAP FIV/FeLV Combo (IDEXX). A total of 58 samples were analysed with conjugate antibody 1:8,000. Samples with discordant results are marked with an asterisk and highlighted in bold. OD: optical density. POS: positive; NEG: negative.

N. sample	FIVCHECK Ab ELISA (Agrolabo)		SNAP FIV/FeLV Combo (IDEXX)	
	OD	Results	FIV	FeLV
1	0.075	NEG	NEG	NEG
2	1.712	POS	POS	POS
3	0.175	NEG	NEG	NEG
4	0.081	NEG	NEG	NEG
5	0.053	NEG	NEG	NEG
6	1.792	POS	POS	NEG
**7***	**0.499**	**POS**	**NEG**	**POS**
8	0.050	NEG	NEG	NEG
9	0.096	NEG	NEG	NEG
10	2.201	POS	POS	NEG
11	1.659	POS	POS	NEG
12	0.227	NEG	NEG	NEG
13	1.895	POS	POS	NEG
14	0.082	NEG	NEG	NEG
15	2.058	POS	POS	NEG
16	2.374	POS	POS	NEG
17	0.110	NEG	NEG	NEG
18	2.039	POS	POS	NEG
19	2.123	POS	POS	NEG
20	2.080	POS	POS	NEG
21	2.105	POS	POS	NEG
22	2.629	POS	POS	NEG
23	0.068	NEG	NEG	NEG
24	0.148	NEG	NEG	POS
25	2.612	POS	POS	NEG
26	0.085	NEG	NEG	NEG
27	0.244	NEG	NEG	NEG
**28***	**0.912**	**POS**	**NEG**	**NEG**
29	2.605	POS	POS	NEG
30	2.613	POS	POS	NEG
31	2.553	POS	POS	NEG
32	2.596	POS	POS	NEG
33	2.556	POS	POS	NEG
34	2.602	POS	POS	NEG
35	0.124	NEG	NEG	POS
36	0.076	NEG	NEG	NEG
37	0.095	NEG	NEG	NEG
**38***	**1.157**	**POS**	**NEG**	**NEG**
**39***	**0.872**	**POS**	**NEG**	**NEG**
40	2.565	POS	POS	NEG
41	2.601	POS	POS	NEG
42	2.577	POS	POS	NEG
43	2.576	POS	POS	NEG
**44***	**0.277**	**DOUBTFUL**	**NEG**	**NEG**
45	0.069	NEG	NEG	POS
46	2.611	POS	POS	NEG
47	2.599	POS	POS	NEG
48	0.177	NEG	NEG	NEG
49	0.096	NEG	NEG	NEG

Continued on the next page

Supplementary Table S2 *(continued)*

N. sample	FIVCHECK Ab ELISA (Agrolabo)		SNAP FIV/FeLV Combo (IDEXX)	
	**OD**	**Results**	**FIV**	**FeLV**
50	2.351	POS	POS	NEG
51	0.162	NEG	NEG	NEG
52	0.081	NEG	NEG	POS
53	0.068	NEG	NEG	NEG
**54***	**0.458**	**POS**	**NEG**	**NEG**
55	0.091	NEG	NEG	NEG
56	0.064	NEG	NEG	NEG
**57***	**0.678**	**POS**	**NEG**	**NEG**
58	0.053	NEG	NEG	NEG

Supplementary Table S3. Results of FIVCHECK Ab ELISA (sample dilution 1:200) against the SNAP FIV/FeLV Combo (IDEXX). A total of 113 samples were analysed with conjugate antibody 1:8,000. Samples doubtful are marked with an asterisk and highlighted in bold. OD: optical density. POS: positive; NEG: negative.

N. sample	FIVCHECK Ab ELISA (Agrolabo)		SNAP FIV/FeLV Combo (IDEXX)	
	**OD**	**Results**	**FIV**	**FeLV**
1	0.055	NEG	NEG	NEG
2	1.462	POS	POS	POS
3	0.104	NEG	NEG	NEG
4	0.050	NEG	NEG	NEG
5	0.043	NEG	NEG	NEG
6	1.511	POS	POS	NEG
7	0.094	NEG	NEG	POS
8	0.083	NEG	NEG	NEG
9	0.057	NEG	NEG	NEG
10	1.619	POS	POS	NEG
11	1.248	POS	POS	NEG
12	0.076	NEG	NEG	NEG
13	1.852	POS	POS	NEG
14	0.047	NEG	NEG	NEG
15	1.884	POS	POS	NEG
16	1.981	POS	POS	NEG
17	0.065	NEG	NEG	NEG
18	1.622	POS	POS	NEG
19	1.695	POS	POS	NEG
20	1.646	POS	POS	NEG
21	1.702	POS	POS	NEG
22	1.753	POS	POS	NEG
23	0.066	NEG	NEG	NEG
24	0.060	NEG	NEG	POS
25	1.594	POS	POS	NEG
26	0.041	NEG	NEG	NEG
27	0.058	NEG	NEG	NEG
**28***	**0.309**	**DOUBTFUL**	**NEG**	**NEG**
29	1.365	POS	POS	NEG
30	1.478	POS	POS	NEG
31	0.985	POS	POS	NEG
32	1.594	POS	POS	NEG
33	2.004	POS	POS	NEG
34	2.261	POS	POS	NEG
35	0.056	NEG	NEG	POS
36	0.048	NEG	NEG	NEG
37	0.042	NEG	NEG	NEG
38	0.300	NEG	NEG	NEG
39	0.260	NEG	NEG	NEG
40	1.640	POS	POS	NEG
41	2.248	POS	POS	NEG
42	1.912	POS	POS	NEG
43	2.187	POS	POS	NEG
44	0.116	NEG	NEG	NEG
45	0.050	NEG	NEG	POS

Continued on the next page

Supplementary Table S3 *(continued)*

N. sample	FIVCHECK Ab ELISA (Agrolabo)		SNAP FIV/FeLV Combo (IDEXX)	
	OD	Results	FIV	FeLV
46	2.072	POS	POS	NEG
47	2.027	POS	POS	NEG
48	0.085	NEG	NEG	NEG
49	0.060	NEG	NEG	NEG
50	1.675	POS	POS	NEG
51	0.081	NEG	NEG	NEG
52	0.055	NEG	NEG	POS
53	0.056	NEG	NEG	NEG
54	0.301	NEG	NEG	NEG
55	0.089	NEG	NEG	NEG
56	0.048	NEG	NEG	NEG
57	0.266	NEG	NEG	NEG
58	0.040	NEG	NEG	NEG
59	0.089	NEG	NEG	NEG
60	0.045	NEG	NEG	NEG
61	0.054	NEG	NEG	NEG
62	0.681	POS	POS	POS
63	0.046	NEG	NEG	NEG
64	0.051	NEG	NEG	NEG
65	0.048	NEG	NEG	NEG
66	0.046	NEG	NEG	NEG
67	0.070	NEG	NEG	NEG
68	0.069	NEG	NEG	NEG
69	0.042	NEG	NEG	NEG
70	0.042	NEG	NEG	NEG
71	0.055	NEG	NEG	NEG
72	0.039	NEG	NEG	NEG
73	0.049	NEG	NEG	NEG
74	0.076	NEG	NEG	NEG
75	0.057	NEG	NEG	NEG
76	0.037	NEG	NEG	NEG
77	0.039	NEG	NEG	NEG
78	0.242	NEG	NEG	NEG
79	0.092	NEG	NEG	NEG
80	0.038	NEG	NEG	NEG
81	0.047	NEG	NEG	NEG
82	0.076	NEG	NEG	NEG
83	2.240	POS	POS	POS
84	3.806	POS	POS	NEG
85	3.439	POS	POS	NEG
86	3.912	POS	POS	NEG
87	3.889	POS	POS	NEG
88	3.781	POS	POS	NEG
89	0.177	NEG	NEG	NEG

Continued on the next page

Supplementary Table S3 *(continued)*

N. sample	FIVCHECK Ab ELISA (Agrolabo)		SNAP FIV/FeLV Combo (IDEXX)	
	OD	Results	FIV	FeLV
90	3.677	POS	POS	NEG
91	3.846	POS	POS	NEG
92	0.106	NEG	NEG	NEG
93	0.139	NEG	NEG	NEG
94	0.113	NEG	NEG	NEG
95	0.136	NEG	NEG	NEG
96	0.127	NEG	NEG	NEG
97	0.096	NEG	NEG	NEG
98	3.409	POS	POS	NEG
99	3.498	POS	POS	NEG
100	0.111	NEG	NEG	NEG
101	0.215	NEG	NEG	NEG
102	0.287	NEG	NEG	NEG
103	0.172	NEG	NEG	NEG
104	0.243	NEG	NEG	NEG
105	0.135	NEG	NEG	NEG
106	0.212	NEG	NEG	NEG
107	0.148	NEG	NEG	NEG
108	0.278	NEG	NEG	NEG
109	0.222	NEG	NEG	NEG
110	0.183	NEG	NEG	NEG
111	0.215	NEG	NEG	NEG
112	0.144	NEG	NEG	NEG
113	0.152	NEG	NEG	NEG

Supplementary Table S4. Conjugate antibody selection in FIVCHECK Ab ELISA. A total of 60 samples diluted 1:200 were analysed with conjugate antibody dilutions 1:8,000 and 1:15,000. The SNAP FIV/FeLV Combo (IDEXX) test was considered as reference. Discordant samples are marked with an asterisk and highlighted in bold. OD: optical density. POS: positive; NEG: negative.

N. sample	SNAP FIV/FeLV Combo (IDEXX)		Conjugate 1:8,000		Conjugate 1:15,000	
	FIV	FeLV	OD	Results	OD	Results
1	POS	POS	1.462	POS	1.353	POS
2	POS	NEG	1.511	POS	1.391	POS
3	POS	NEG	1.619	POS	1.169	POS
4	POS	NEG	1.248	POS	1.301	POS
5	POS	NEG	1.852	POS	1.521	POS
6	POS	NEG	1.884	POS	1.363	POS
7	POS	NEG	1.981	POS	1.650	POS
8	POS	NEG	1.622	POS	1.178	POS
9	POS	NEG	1.695	POS	1.231	POS
10	POS	NEG	1.646	POS	1.188	POS
11	POS	NEG	1.702	POS	1.256	POS
12	POS	NEG	1.753	POS	1.234	POS
13	POS	NEG	1.594	POS	1.161	POS
14	POS	NEG	1.365	POS	0.986	POS
15	POS	NEG	1.478	POS	1.081	POS
16	POS	NEG	0.985	POS	1.339	POS
17	POS	NEG	1.594	POS	1.111	POS
18	POS	NEG	2.004	POS	1.410	POS
19	POS	NEG	2,261	POS	1.656	POS
20	POS	NEG	1.640	POS	1.247	POS
21	POS	NEG	2.248	POS	1.637	POS
22	POS	NEG	1.912	POS	1.389	POS
23	POS	NEG	2.187	POS	1.606	POS
24	POS	NEG	2.072	POS	1.598	POS
25	POS	NEG	2.027	POS	1.528	POS
26	POS	NEG	1.675	POS	1.456	POS
27	POS	POS	0.681	POS	0.493	POS
28	POS	POS	2.240	POS	1.675	POS
29	NEG	NEG	0.055	NEG	0.048	NEG
30	NEG	NEG	0.104	NEG	0.034	NEG
31	NEG	NEG	0.050	NEG	0.041	NEG
32	NEG	NEG	0.043	NEG	0.042	NEG
33	NEG	POS	0.094	NEG	0.080	NEG
34	NEG	NEG	0.083	NEG	0.066	NEG
35	NEG	NEG	0.057	NEG	0.047	NEG
36	NEG	NEG	0.076	NEG	0.070	NEG
37	NEG	NEG	0.047	NEG	0.050	NEG
38	NEG	NEG	0.065	NEG	0.107	NEG
39	NEG	NEG	0.066	NEG	0.058	NEG
40	NEG	POS	0.060	NEG	0.053	NEG
41	NEG	NEG	0.041	NEG	0.044	NEG
42	NEG	NEG	0.058	NEG	0.043	NEG
43	NEG	POS	0.056	NEG	0.058	NEG
44	NEG	NEG	0.048	NEG	0.060	NEG
45	NEG	NEG	0.042	NEG	0.038	NEG
46	NEG	NEG	0.116	NEG	0.111	NEG
47	NEG	POS	0.050	NEG	0.032	NEG

Continued on the next page

Supplementary Table S4 *(continued)*

N. sample	SNAP FIV/FeLV Combo (IDEXX)		Conjugate 1:8,000		Conjugate 1:15,000	
	**FIV**	**FeLV**	**OD**	**Results**	**OD**	**Results**
48	NEG	NEG	0.085	NEG	0.096	NEG
49	NEG	NEG	0.060	NEG	0.059	NEG
50	NEG	NEG	0.081	NEG	0.059	NEG
51	NEG	POS	0.055	NEG	0.052	NEG
52	NEG	NEG	0.056	NEG	0.045	NEG
**53***	**NEG**	**NEG**	**0.309**	**DOUBTFUL**	**0.242**	**NEG**
54	NEG	NEG	0.300	NEG	0.261	NEG
55	NEG	NEG	0.260	NEG	0.252	NEG
56	NEG	NEG	0.301	NEG	0.135	NEG
57	NEG	NEG	0.266	NEG	0.185	NEG
58	NEG	NEG	0.287	NEG	0.185	NEG
59	NEG	NEG	0.243	NEG	0.070	NEG
60	NEG	NEG	0.278	NEG	0.167	NEG

Supplementary Table S5. Validation results of FIVCHECK Ab ELISA against the reference test SNAP FIV/FeLV Combo (IDEXX). Total number of samples analysed: 115. Doubtful samples are marked with an asterisk and highlighted in bold. OD: optical density. POS: positive; NEG: negative.

N. sample	FIVCHECK Ab ELISA (Agrolabo)		SNAP FIV/FeLV Combo (IDEXX)	
	OD	Results	FIV	FeLV
1	0.048	NEG	NEG	NEG
2	1.353	POS	POS	POS
3	0.034	NEG	NEG	NEG
4	0.041	NEG	NEG	NEG
5	0.042	NEG	NEG	NEG
6	1.391	POS	POS	NEG
7	0.080	NEG	NEG	POS
8	0.066	NEG	NEG	NEG
9	0.047	NEG	NEG	NEG
10	1.169	POS	POS	NEG
11	1.301	POS	POS	NEG
12	0.070	NEG	NEG	NEG
13	1.521	POS	POS	NEG
14	0.050	NEG	NEG	NEG
15	1.363	POS	POS	NEG
16	1.650	POS	POS	NEG
17	0.107	NEG	NEG	NEG
18	1.178	POS	POS	NEG
19	1.231	POS	POS	NEG
20	1.188	POS	POS	NEG
21	1.256	POS	POS	NEG
22	1.234	POS	POS	NEG
23	0.058	NEG	NEG	NEG
24	0.053	NEG	NEG	POS
25	1.161	POS	POS	NEG
26	0.044	NEG	NEG	NEG
27	0.043	NEG	NEG	NEG
**28***	**0.242**	**DOUBTFUL**	**NEG**	**NEG**
29	0.986	POS	POS	NEG
30	1.081	POS	POS	NEG
31	1.339	POS	POS	NEG
32	1.111	POS	POS	NEG
33	1.410	POS	POS	NEG
34	1.656	POS	POS	NEG
35	0.058	NEG	NEG	POS
36	0.060	NEG	NEG	NEG
37	0.038	NEG	NEG	NEG
**38***	**0.261**	**DOUBTFUL**	**NEG**	**NEG**
**39***	**0.252**	**DOUBTFUL**	**NEG**	**NEG**
40	1.247	POS	POS	NEG
41	1.637	POS	POS	NEG
42	1.389	POS	POS	NEG
43	1.606	POS	POS	NEG
44	0.111	NEG	NEG	NEG
45	0.032	NEG	NEG	POS
46	1.598	POS	POS	NEG
47	1.528	POS	POS	NEG
48	0.096	NEG	NEG	NEG
49	0.059	NEG	NEG	NEG
50	1.456	POS	POS	NEG

Continued on the next page

Supplementary Table S5 *(continued)*

N. sample	FIVCHECK Ab ELISA (Agrolabo)		SNAP FIV/FeLV Combo (IDEXX)	
	OD	Results	FIV	FeLV
51	0.059	NEG	NEG	NEG
52	0.052	NEG	NEG	POS
53	0.045	NEG	NEG	NEG
54	0.135	NEG	NEG	NEG
55	0.061	NEG	NEG	NEG
56	0.045	NEG	NEG	NEG
57	0.185	NEG	NEG	NEG
58	0.047	NEG	NEG	NEG
59	0.077	NEG	NEG	NEG
60	0.049	NEG	NEG	NEG
61	0.044	NEG	NEG	NEG
62	0.493	POS	POS	POS
63	0.043	NEG	NEG	NEG
64	0.048	NEG	NEG	NEG
65	0.048	NEG	NEG	NEG
66	0.041	NEG	NEG	NEG
67	0.041	NEG	NEG	NEG
68	0.065	NEG	NEG	NEG
69	0.045	NEG	NEG	NEG
70	0.049	NEG	NEG	NEG
71	0.049	NEG	NEG	NEG
72	0.041	NEG	NEG	NEG
73	0.041	NEG	NEG	NEG
74	0.047	NEG	NEG	NEG
75	0.041	NEG	NEG	NEG
76	0.046	NEG	NEG	NEG
77	0.035	NEG	NEG	NEG
78	0.108	NEG	NEG	NEG
79	0.105	NEG	NEG	NEG
80	0.039	NEG	NEG	NEG
81	0.050	NEG	NEG	NEG
82	0.068	NEG	NEG	NEG
83	1.675	POS	POS	POS
84	2.778	POS	POS	NEG
85	2.370	POS	POS	NEG
86	2.808	POS	POS	NEG
87	3.344	POS	POS	NEG
88	3.407	POS	POS	NEG
89	0.134	NEG	NEG	NEG
90	2.545	POS	POS	NEG
91	2.927	POS	POS	NEG
92	0.076	NEG	NEG	NEG
93	0.132	NEG	NEG	NEG
94	0.110	NEG	NEG	NEG
95	0.098	NEG	NEG	NEG
96	0.076	NEG	NEG	NEG
97	0.075	NEG	NEG	NEG
98	2.141	POS	POS	NEG
99	2.133	POS	POS	NEG
100	0.075	NEG	NEG	NEG

Continued on the next page

Supplementary Table S5 *(continued)*

N. sample	FIVCHECK Ab ELISA (Agrolabo)		SNAP FIV/FeLV Combo (IDEXX)	
	**OD**	**Results**	**FIV**	**FeLV**
101	0.132	NEG	NEG	NEG
102	0.185	NEG	NEG	NEG
103	0.197	NEG	NEG	NEG
104	0.070	NEG	NEG	NEG
105	0.055	NEG	NEG	NEG
106	0.058	NEG	NEG	NEG
107	0.056	NEG	NEG	NEG
108	0.167	NEG	NEG	NEG
109	0.056	NEG	NEG	NEG
110	0.054	NEG	NEG	NEG
111	0.054	NEG	NEG	NEG
112	0.057	NEG	NEG	NEG
113	0.053	NEG	NEG	NEG
114	0.070	NEG	NEG	NEG
115	2.318	POS	POS	NEG

Supplementary Table S6. Cut-off determination. Sp: Specificity; Se: Sensitivity; J: Jouden's index.

N. Cut-off	OD cut-off	Sp	1 - Sp	Se	Se-Sp	J	VPP	VPN	Accuracy	LR +	LR -	J max
1	0.030	0.000	1.000	1.000	1.000	0.000	0.272	-	0.272	1.000	-	1.000
2	0.040	0.038	0.962	1.000	0.962	0.038	0.250	1.000	0.272	1.040	0.000	
3	0.050	0.093	0.907	1.000	0.907	0.093	0.292	1.000	0.340	1.103	0.000	
4	0.060	0.267	0.733	1.000	0.733	0.267	0.337	1.000	0.466	1.364	0.000	
5	0.070	0.400	0.600	1.000	0.600	0.400	0.384	1.000	0.563	1.667	0.000	
6	0.080	0.547	0.453	1.000	0.453	0.547	0.452	1.000	0.670	2.206	0.000	
7	0.090	0.613	0.387	1.000	0.387	0.613	0.491	1.000	0.718	2.586	0.000	
8	0.100	0.640	0.360	1.000	0.360	0.640	0.509	1.000	0.738	2.778	0.000	
9	0.110	0.707	0.293	1.000	0.293	0.707	0.560	1.000	0.786	3.409	0.000	
10	0.120	0.760	0.240	1.000	0.240	0.760	0.609	1.000	0.825	4.167	0.000	
11	0.130	0.813	0.187	1.000	0.187	0.813	0.667	1.000	0.864	5.357	0.000	
12	0.140	0.853	0.147	1.000	0.147	0.853	0.718	1.000	0.893	6.818	0.000	
13	0.150	0.893	0.107	1.000	0.107	0.893	0.778	1.000	0.922	9.375	0.000	
14	0.160	0.893	0.107	1.000	0.107	0.893	0.778	1.000	0.922	9.375	0.000	
15	0.170	0.933	0.067	1.000	0.067	0.933	0.848	1.000	0.951	15.000	0.000	
16	0.180	0.933	0.067	1.000	0.067	0.933	0.848	1.000	0.951	15.000	0.000	
17	0.190	0.960	0.040	1.000	0.040	0.960	0.903	1.000	0.971	25.000	0.000	
18	0.200	0.960	0.040	1.000	0.040	0.960	0.903	1.000	0.971	25.000	0.000	
19	0.210	0.960	0.040	1.000	0.040	0.960	0.903	1.000	0.971	25.000	0.000	
20	0.220	0.960	0.040	1.000	0.040	0.960	0.903	1.000	0.971	25.000	0.000	
21	0.230	0.960	0.040	1.000	0.040	0.960	0.903	1.000	0.971	25.000	0.000	
22	0.240	0.960	0.040	1.000	0.040	0.960	0.903	1.000	0.971	25.000	0.000	
23	0.250	0.973	0.027	1.000	0.027	0.973	0.933	1.000	0.981	37.500	0.000	
24	0.260	0.987	0.013	1.000	0.013	0.987	0.966	1.000	0.990	75.000	0.000	
25	0.270	1.000	0.000	1.000	0.000	1.000	1.000	1.000	1.000	-	0.000	
26	0.280	1.000	0.000	1.000	0.000	1.000	1.000	1.000	1.000	-	0.000	
27	0.290	1.000	0.000	1.000	0.000	1.000	1.000	1.000	1.000	-	0.000	
28	0.300	1.000	0.000	1.000	0.000	1.000	1.000	1.000	1.000	-	0.000	
29	0.310	1.000	0.000	1.000	0.000	1.000	1.000	1.000	1.000	-	0.000	
30	0.320	1.000	0.000	1.000	0.000	1.000	1.000	1.000	1.000	-	0.000	
31	0.330	1.000	0.000	1.000	0.000	1.000	1.000	1.000	1.000	-	0.000	
32	0.340	1.000	0.000	1.000	0.000	1.000	1.000	1.000	1.000	-	0.000	
33	0.350	1.000	0.000	1.000	0.000	1.000	1.000	1.000	1.000	-	0.000	
34	0.360	1.000	0.000	1.000	0.000	1.000	1.000	1.000	1.000	-	0.000	
35	0.370	1.000	0.000	1.000	0.000	1.000	1.000	1.000	1.000	-	0.000	
36	0.380	1.000	0.000	1.000	0.000	1.000	1.000	1.000	1.000	-	0.000	
37	0.390	1.000	0.000	1.000	0.000	1.000	1.000	1.000	1.000	-	0.000	
38	0.400	1.000	0.000	1.000	0.000	1.000	1.000	1.000	1.000	-	0.000	
39	0.410	1.000	0.000	1.000	0.000	1.000	1.000	1.000	1.000	-	0.000	
40	0.420	1.000	0.000	1.000	0.000	1.000	1.000	1.000	1.000	-	0.000	
41	0.430	1.000	0.000	1.000	0.000	1.000	1.000	1.000	1.000	-	0.000	
42	0.440	1.000	0.000	1.000	0.000	1.000	1.000	1.000	1.000	-	0.000	
43	0.450	1.000	0.000	1.000	0.000	1.000	1.000	1.000	1.000	-	0.000	
44	0.460	28.000	75.000	28.000	0.000	0.000	75.000	1.000	0.000	1.000	0.000	
45	0.470	28.000	75.000	28.000	0.000	0.000	75.000	1.000	0.000	1.000	0.000	
46	0.480	28.000	75.000	28.000	0.000	0.000	75.000	1.000	0.000	1.000	0.000	
47	0.490	28.000	75.000	28.000	0.000	0.000	75.000	1.000	0.000	1.000	0.000	
48	0.500	28.000	75.000	28.000	0.000	0.000	75.000	1.000	0.000	1.000	0.000	
49	0.510	28.000	75.000	28.000	0.000	0.000	75.000	1.000	0.000	1.000	0.000	
50	0.520	28.000	75.000	28.000	0.000	0.000	75.000	1.000	0.000	1.000	0.000	
51	0.530	28.000	75.000	28.000	0.000	0.000	75.000	1.000	0.000	1.000	0.000	
52	0.540	27.000	76.000	27.000	0.000	1.000	75.000	1.000	0.000	0.964	-0.036	
53	0.550	27.000	76.000	27.000	0.000	1.000	75.000	1.000	0.000	0.964	-0.036	
54	0.560	27.000	76.000	27.000	0.000	1.000	75.000	1.000	0.000	0.964	-0.036	

Continued on the next page

Supplementary Table S6 *(continued)*

N. Cut-off	OD cut-off	Sp	1 - Sp	Se	Se-Sp	J	VPP	VPN	Accuracy	LR +	LR -
55	0.570	27.000	76.000	27.000	0.000	1.000	75.000	1.000	0.000	0.964	-0.036
56	0.580	27.000	76.000	27.000	0.000	1.000	75.000	1.000	0.000	0.964	-0.036
57	0.590	27.000	76.000	27.000	0.000	1.000	75.000	1.000	0.000	0.964	-0.036
58	0.600	27.000	76.000	27.000	0.000	1.000	75.000	1.000	0.000	0.964	-0.036
59	0.610	27.000	76.000	27.000	0.000	1.000	75.000	1.000	0.000	0.964	-0.036
60	0.620	27.000	76.000	27.000	0.000	1.000	75.000	1.000	0.000	0.964	-0.036
61	0.630	27.000	76.000	27.000	0.000	1.000	75.000	1.000	0.000	0.964	-0.036
62	0.640	27.000	76.000	27.000	0.000	1.000	75.000	1.000	0.000	0.964	-0.036
63	0.650	27.000	76.000	27.000	0.000	1.000	75.000	1.000	0.000	0.964	-0.036
64	0.660	27.000	76.000	27.000	0.000	1.000	75.000	1.000	0.000	0.964	-0.036
65	0.670	27.000	76.000	27.000	0.000	1.000	75.000	1.000	0.000	0.964	-0.036
66	0.680	27.000	76.000	27.000	0.000	1.000	75.000	1.000	0.000	0.964	-0.036
67	0.690	27.000	76.000	27.000	0.000	1.000	75.000	1.000	0.000	0.964	-0.036
68	0.700	27.000	76.000	27.000	0.000	1.000	75.000	1.000	0.000	0.964	-0.036
69	0.710	27.000	76.000	27.000	0.000	1.000	75.000	1.000	0.000	0.964	-0.036
70	0.720	27.000	76.000	27.000	0.000	1.000	75.000	1.000	0.000	0.964	-0.036
71	0.730	27.000	76.000	27.000	0.000	1.000	75.000	1.000	0.000	0.964	-0.036
72	0.740	27.000	76.000	27.000	0.000	1.000	75.000	1.000	0.000	0.964	-0.036
73	0.750	27.000	76.000	27.000	0.000	1.000	75.000	1.000	0.000	0.964	-0.036
74	0.760	27.000	76.000	27.000	0.000	1.000	75.000	1.000	0.000	0.964	-0.036
75	0.770	27.000	76.000	27.000	0.000	1.000	75.000	1.000	0.000	0.964	-0.036
76	0.780	27.000	76.000	27.000	0.000	1.000	75.000	1.000	0.000	0.964	-0.036
77	0.790	27.000	76.000	27.000	0.000	1.000	75.000	1.000	0.000	0.964	-0.036
78	0.800	27.000	76.000	27.000	0.000	1.000	75.000	1.000	0.000	0.964	-0.036
79	0.810	27.000	76.000	27.000	0.000	1.000	75.000	1.000	0.000	0.964	-0.036
80	0.820	27.000	76.000	27.000	0.000	1.000	75.000	1.000	0.000	0.964	-0.036
81	0.830	27.000	76.000	27.000	0.000	1.000	75.000	1.000	0.000	0.964	-0.036
82	0.840	27.000	76.000	27.000	0.000	1.000	75.000	1.000	0.000	0.964	-0.036
83	0.850	27.000	76.000	27.000	0.000	1.000	75.000	1.000	0.000	0.964	-0.036
84	0.860	27.000	76.000	27.000	0.000	1.000	75.000	1.000	0.000	0.964	-0.036
85	0.870	27.000	76.000	27.000	0.000	1.000	75.000	1.000	0.000	0.964	-0.036
86	0.880	27.000	76.000	27.000	0.000	1.000	75.000	1.000	0.000	0.964	-0.036
87	0.890	27.000	76.000	27.000	0.000	1.000	75.000	1.000	0.000	0.964	-0.036
88	0.900	27.000	76.000	27.000	0.000	1.000	75.000	1.000	0.000	0.964	-0.036
89	0.910	27.000	76.000	27.000	0.000	1.000	75.000	1.000	0.000	0.964	-0.036
90	0.920	27.000	76.000	27.000	0.000	1.000	75.000	1.000	0.000	0.964	-0.036
91	0.930	27.000	76.000	27.000	0.000	1.000	75.000	1.000	0.000	0.964	-0.036
92	0.940	27.000	76.000	27.000	0.000	1.000	75.000	1.000	0.000	0.964	-0.036
93	0.950	27.000	76.000	27.000	0.000	1.000	75.000	1.000	0.000	0.964	-0.036
94	0.960	27.000	76.000	27.000	0.000	1.000	75.000	1.000	0.000	0.964	-0.036
95	0.970	27.000	76.000	27.000	0.000	1.000	75.000	1.000	0.000	0.964	-0.036
96	0.980	27.000	76.000	27.000	0.000	1.000	75.000	1.000	0.000	0.964	-0.036
97	0.990	27.000	76.000	27.000	0.000	1.000	75.000	1.000	0.000	0.964	-0.036
98	1.000	27.000	76.000	27.000	0.000	1.000	75.000	1.000	0.000	0.964	-0.036
99	1.010	27.000	76.000	27.000	0.000	1.000	75.000	1.000	0.000	0.964	-0.036
100	1.020	27.000	76.000	27.000	0.000	1.000	75.000	1.000	0.000	0.964	-0.036
101	1.030	27.000	76.000	27.000	0.000	1.000	75.000	1.000	0.000	0.964	-0.036
102	1.040	27.000	76.000	27.000	0.000	1.000	75.000	1.000	0.000	0.964	-0.036

Supplementary Table S7. Reproducibility study. ELISA tests were performed in duplicate, twice a day, for 14 consecutive days (56 tests for each sample). PC1: positive control anti-His HRP antibody (1:300); PC2: a positive control anti-His HRP antibody (1:60,000); NC: negative control; sample 121 (negative), sample 3 (positive), sample 7 (positive).

**Samples**	**Day 1**				**Day 2**				**Day 3**				**Day 4**				**Day 5**			
	**OD 1**	**OD 2**	**OD 3**	**OD 4**	**OD 1**	**OD 2**	**OD 3**	**OD 4**	**OD 1**	**OD 2**	**OD 3**	**OD 4**	**OD 1**	**OD 2**	**OD 3**	**OD 4**	**OD 1**	**OD 2**	**OD 3**	**OD 4**
PC1	2.621	2.627	2.644	2.637	2.650	2.665	2.631	2.646	2.680	2.657	2.682	2.672	2.666	2.692	2.666	2.692	2.734	2.736	2.706	2.676
PC2	3.121	3.129	3.166	3.169	3.117	3.199	3.158	3.167	3.141	3.149	3.169	3.162	3.151	3.158	3.149	3.141	3.188	3.181	3.251	3.258
NC	0.035	0.034	0.036	0.036	0.033	0.033	0.036	0.038	0.035	0.034	0.035	0.034	0.035	0.035	0.035	0.035	0.036	0.036	0.033	0.036
Sample 121 (-)	0.044	0.044	0.045	0.049	0.033	0.033	0.046	0.047	0.036	0.034	0.038	0.040	0.042	0.041	0.042	0.041	0.040	0.039	0.037	0.043
Sample 3 (+)	1.501	1.510	1.520	1.523	1.530	1.528	1.514	1.520	1.511	1.517	1.509	1.516	1.507	1.507	1.510	1.507	1.500	1.512	1.526	1.520
Sample 7 (+)	1.730	1.730	1.659	1.663	1.800	1.808	1.726	1.674	1.767	1.781	1.765	1.766	1.764	1.767	1.765	1.767	1.707	1.759	1.715	1.774

**Samples**	**Day 6**				**Day 7**				**Day 8**				**Day 9**				**Day 10**			
	**OD 1**	**OD 2**	**OD 3**	**OD 4**	**OD 1**	**OD 2**	**OD 3**	**OD 4**	**OD 1**	**OD 2**	**OD 3**	**OD 4**	**OD 1**	**OD 2**	**OD 3**	**OD 4**	**OD 1**	**OD 2**	**OD 3**	**OD 4**
PC1	2.740	2.732	2.703	2.732	2.703	2.732	2.703	2.732	2.703	2.703	2.755	2.706	2.786	2.707	2.762	2.688	2.693	2.695	2.709	2.691
PC2	3.121	3.141	3.146	3.141	3.146	3.141	3.146	3.141	3.146	3.146	3.208	3.211	3.198	3.201	3.146	3.142	3.112	3.115	3.153	3.149
NC	0.033	0.035	0.035	0.035	0.035	0.035	0.035	0.035	0.035	0.035	0.032	0.035	0.034	0.036	0.034	0.036	0.034	0.039	0.034	0.037
Sample 121 (-)	0.037	0.040	0.038	0.040	0.038	0.040	0.038	0.040	0.038	0.038	0.036	0.040	0.038	0.039	0.038	0.041	0.036	0.034	0.036	0.041
Sample 3 (+)	1.520	1.500	1.514	1.500	1.514	1.500	1.514	1.500	1.514	1.514	1.521	1.513	1.521	1.511	1.520	1.511	1.485	1.437	1.496	1.437
Sample 7 (+)	1.709	1.707	1.769	1.707	1.769	1.707	1.769	1.707	1.769	1.769	1.708	1.776	1.712	1.775	1.711	1.776	1.742	1.740	1.753	1.733

**Samples**	**Day 11**				**Day 12**				**Day 13**				**Day 14**			
	**OD 1**	**OD 2**	**OD 3**	**OD 4**	**OD 1**	**OD 2**	**OD 3**	**OD 4**	**OD 1**	**OD 2**	**OD 3**	**OD 4**	**OD 1**	**OD 2**	**OD 3**	**OD 4**
PC1	2.706	2.689	2.689	2.685	2.698	2.689	2.705	2.695	2.703	2.676	2.725	2.700	2.725	2.700	2.783	2.696
PC2	3.203	3.199	3.251	3.254	3.131	3.138	3.163	3.158	3.124	3.121	3.171	3.174	3.125	3.131	3.161	3.169
NC	0.034	0.037	0.034	0.037	0.033	0.036	0.033	0.038	0.033	0.037	0.033	0.037	0.033	0.037	0.033	0.037
Sample 121 (-)	0.034	0.039	0.034	0.038	0.038	0.041	0.039	0.037	0.037	0.039	0.039	0.041	0.036	0.041	0.038	0.038
Sample 3 (+)	1.491	1.448	1.493	1.448	1.497	1.441	1.495	1.439	1.491	1.437	1.498	1.431	1.498	1.431	1.490	1.427
Sample 7 (+)	1.756	1.735	1.759	1.739	1.759	1.742	1.761	1.744	1.760	1.745	1.764	1.742	1.764	1.742	1.755	1.747

Supplementary Table S8. Intra- and inter-assay coefficient of variation of FIVCHECK Ab ELISA. PC1: positive control anti-His HRP antibody (1:300); PC2: a positive control anti-His HRP antibody (1:60,000); NC: negative control; sample 121 (negative), sample 3 (positive), sample 7 (positive). %CV: coefficient of variation (%); MIN: minimum %CV; MAX: maximum %CV; n: number of assays.

Day of analysis	N. assays	Intra-assay %CV						Inter-assay %CV					
		PC1	PC2	NC	Sample 121 (-)	Sample 3 (+)	Sample 7 (+)	PC1	PC2	NC	Sample 121 (-)	Sample 3 (+)	Sample 7 (+)
Day 1	1	0.162	0.181	2.050	0.000	0.423	0.000	0.175	0.124	1.025	3.009	0.281	0.085
	2	0.187	0.067	0.000	6.018	0.139	0.170						
Day 2	3	0.399	1.836	0.000	0.000	0.092	0.314	0.401	1.019	1.911	0.760	0.186	1.238
	4	0.402	0.201	3.822	1.521	0.280	2.163						
Day 3	5	0.609	0.180	2.050	4.041	0.280	0.558	0.437	0.168	2.050	3.833	0.304	0.299
	6	0.264	0.156	2.050	3.626	0.327	0.040						
Day 4	7	0.686	0.157	0.000	1.704	0.000	0.120	0.686	0.168	0.000	1.704	0.070	0.100
	8	0.686	0.180	0.000	1.704	0.141	0.080						
Day 5	9	0.052	0.155	0.000	1.790	0.563	2.122	0.420	0.154	3.074	6.198	0.421	2.257
	10	0.788	0.152	6.149	10.607	0.279	2.391						
Day 6	11	0.259	0.339	6.149	3.722	0.093	2.759	0.350	0.192	4.130	4.616	0.209	2.698
	12	0.441	0.045	2.111	5.510	0.326	2.638						
Day 7	13	0.755	0.112	0.000	3.626	0.657	2.522	1.012	0.089	3.166	5.535	0.515	2.641
	14	1.269	0.066	6.332	7.443	0.373	2.760						
Day 8	15	1.925	0.045	2.111	1.837	0.604	2.757	1.735	0.044	3.135	1.887	0.489	2.697
	16	1.544	0.044	4.159	1.937	0.373	2.636						
Day 9	17	2.034	0.066	4.041	1.837	0.466	2.555	1.977	0.078	4.041	3.604	0.443	2.596
	18	1.920	0.090	4.041	5.370	0.420	2.636						
Day 10	19	0.052	0.068	9.686	4.041	2.323	0.081	0.262	0.079	7.831	6.612	2.584	0.446
	20	0.471	0.090	5.976	9.183	2.845	0.811						
Day 11	21	0.446	0.088	5.976	9.686	2.069	0.851	0.275	0.077	5.976	8.772	2.116	0.830
	22	0.105	0.065	5.976	7.857	2.164	0.809						
Day 12	23	0.236	0.158	6.149	5.370	2.696	0.687	0.249	0.135	8.054	4.546	2.697	0.686
	24	0.262	0.112	9.959	3.722	2.699	0.686						
Day 13	25	0.710	0.068	8.081	3.722	2.608	0.605	0.681	0.067	8.081	3.629	2.922	0.746
	26	0.652	0.067	8.081	3.536	3.235	0.887						
Day 14	27	0.652	0.136	8.081	9.183	3.235	0.887	1.449	0.157	8.081	4.592	3.145	0.605
	28	2.246	0.179	8.081	0.000	3.054	0.323						
**MIN**		**0.052**	**0.044**	**0.000**	**0.000**	**0.000**	**0.000**	**0.175**	**0.044**	**0.000**	**0.760**	**0.070**	**0.085**
**MAX**		**2.246**	**1.836**	**9.959**	**10.607**	**3.235**	**2.760**	**1.977**	**1.019**	**8.081**	**8.772**	**3.145**	**2.698**

Supplementary Table S9. Comparison results of FIVCHECK Ab ELISA, INgezim FIV and VetLine. FIVCHECK Ab ELISA was compared with INgezim FIV ELISA (Gold Standard Diagnostics, GSD) and VetLine FIV ELISA (NovaTec). Total number of samples analyzed: 103. Discordant samples are marked with an asterisk and highlighted in bold. OD: optical density. POS: positive; NEG: negative.

N. sample	INgezim FIV ELISA (GSD)		VetLine FIV (NovaTec)		FIVCHECK Ab ELISA (Agrolabo)	
	OD	Results	OD	Results	OD	Results
1	0.048	NEG	0.258	NEG	0.048	NEG
2	1.719	POS	1.344	POS	1.353	POS
3	0.066	NEG	0.194	NEG	0.034	NEG
**4***	**0.063**	**NEG**	**0.621**	**DOUBTFUL**	**0.041**	**NEG**
5	0.048	NEG	0.202	NEG	0.042	NEG
6	1.820	POS	2.008	POS	1.391	POS
7	0.317	NEG	0.084	NEG	0.080	NEG
**8***	**0.076**	**NEG**	**1.311**	**POS**	**0.066**	**NEG**
**9***	**0.089**	**NEG**	**0.890**	**POS**	**0.047**	**NEG**
10	2.357	POS	2.131	POS	1.169	POS
**11***	**1.981**	**POS**	**0.373**	**NEG**	**1.301**	**POS**
12	0.047	NEG	0.372	NEG	0.070	NEG
13	2.159	POS	2.319	POS	1.521	POS
14	0.044	NEG	0.265	NEG	0.050	NEG
15	1.991	POS	2.280	POS	1.363	POS
16	1.787	POS	2.294	POS	1.650	POS
17	0.041	NEG	0.517	NEG	0.107	NEG
18	2.220	POS	2.185	POS	1.178	POS
19	2.184	POS	2.186	POS	1.231	POS
20	2.284	POS	2.116	POS	1.188	POS
21	2.432	POS	1.933	POS	1.256	POS
22	2.527	POS	2.379	POS	1.234	POS
23	0.046	NEG	0.128	NEG	0.058	NEG
24	0.216	NEG	0.156	NEG	0.053	NEG
25	2.347	POS	2.258	POS	1.161	POS
26	0.048	NEG	0.127	NEG	0.044	NEG
27	0.046	NEG	0.072	NEG	0.043	NEG
28	0.086	NEG	0.474	NEG	0.242	NEG
29	2.346	POS	2.027	POS	0.986	POS
30	1.605	POS	2.196	POS	1.081	POS
31	1.213	POS	1.705	POS	1.339	POS
32	1.603	POS	2.240	POS	1.111	POS
33	1.524	POS	2.034	POS	1.410	POS
34	2.302	POS	2.169	POS	1.656	POS
35	0.157	NEG	0.209	NEG	0.058	NEG
**36***	**0.044**	**NEG**	**0.707**	**POS**	**0.060**	**NEG**
37	0.054	NEG	0.064	NEG	0.038	NEG
**38***	**0.178**	**NEG**	**0.791**	**POS**	**0.261**	**NEG**
39	0.069	NEG	0.089	NEG	0.252	NEG
40	1.785	POS	1.837	POS	1.247	POS
41	2.459	POS	2.315	POS	1.637	POS
42	1.462	POS	2.139	POS	1.389	POS
43	2.459	POS	2.292	POS	1.606	POS
44	0.049	NEG	0.194	NEG	0.111	NEG
45	0.121	NEG	0.187	NEG	0.032	NEG
46	2.421	POS	2.058	POS	1.598	POS
47	2.225	POS	2.254	POS	1.528	POS
**48***	**0.059**	**NEG**	**0.974**	**POS**	**0.096**	**NEG**
**49***	**0.068**	**NEG**	**0.951**	**POS**	**0.087**	**NEG**
50	0.064	NEG	0.277	NEG	0.059	NEG

Continued on the next page

Supplementary Table S9 *(continued)*

N. sample	INgezim FIV ELISA (GSD)		VetLine FIV (NovaTec)		FIVCHECK Ab ELISA (Agrolabo)	
	OD	Results	OD	Results	OD	Results
51	2.066	POS	1.776	POS	1.456	POS
52	0.096	NEG	0.119	NEG	0.059	NEG
53	0.066	NEG	0.072	NEG	0.052	NEG
54	0.052	NEG	0.156	NEG	0.045	NEG
55	0.066	NEG	0.094	NEG	0.135	NEG
56	0.064	NEG	0.108	NEG	0.061	NEG
57	0.049	NEG	0.090	NEG	0.045	NEG
58	0.094	NEG	0.206	NEG	0.185	NEG
59	0.046	NEG	0.089	NEG	0.047	NEG
60	0.039	NEG	0.061	NEG	0.046	NEG
61	0.060	NEG	0.084	NEG	0.048	NEG
62	0.043	NEG	0.066	NEG	0.040	NEG
63	0.039	NEG	0.087	NEG	0.048	NEG
64	0.044	NEG	0.067	NEG	0.077	NEG
65	0.042	NEG	0.071	NEG	0.049	NEG
66	0.068	NEG	0.138	NEG	0.044	NEG
**67***	**0.822**	**POS**	**0.454**	**NEG**	**0.493**	**POS**
68	0.059	NEG	0.084	NEG	0.043	NEG
69	0.062	NEG	0.080	NEG	0.048	NEG
70	0.044	NEG	0.064	NEG	0.048	NEG
71	0.072	NEG	0.154	NEG	0.041	NEG
72	0.060	NEG	0.227	NEG	0.041	NEG
73	0.066	NEG	0.111	NEG	0.065	NEG
74	0.059	NEG	0.073	NEG	0.045	NEG
75	0.050	NEG	0.134	NEG	0.049	NEG
76	0.099	NEG	0.169	NEG	0.049	NEG
77	0.050	NEG	0.071	NEG	0.041	NEG
78	0.087	NEG	0.085	NEG	0.041	NEG
79	0.249	NEG	0.177	NEG	0.047	NEG
80	0.118	NEG	0.120	NEG	0.041	NEG
81	0.043	NEG	0.127	NEG	0.046	NEG
82	0.061	NEG	0.199	NEG	0.035	NEG
83	0.093	NEG	0.089	NEG	0.108	NEG
84	0.114	NEG	0.110	NEG	0.105	NEG
85	0.084	NEG	0.069	NEG	0.039	NEG
86	0.043	NEG	0.101	NEG	0.050	NEG
87	0.109	NEG	0.119	NEG	0.068	NEG
88	0.067	NEG	0.071	NEG	0.047	NEG
89	0.183	NEG	0.155	NEG	0.041	NEG
90	0.098	NEG	0.092	NEG	0.054	NEG
91	0.043	NEG	0.090	NEG	0.040	NEG
92	0.063	NEG	0.089	NEG	0.050	NEG
93	0.068	NEG	0.061	NEG	0.030	NEG
94	0.064	NEG	0.084	NEG	0.043	NEG
95	0.188	NEG	0.066	NEG	0.044	NEG
96	0.089	NEG	0.087	NEG	0.049	NEG
97	0.065	NEG	0.067	NEG	0.048	NEG
98	0.076	NEG	0.071	NEG	0.031	NEG
99	2.467	POS	2.287	POS	1.675	POS
100	0.046	NEG	0.137	NEG	0.037	NEG
101	0.050	NEG	0.084	NEG	0.036	NEG
102	0.180	NEG	0.109	NEG	0.182	NEG
103	0.096	NEG	0.080	NEG	0.094	NEG

Supplementary Table S10. Accelerated stability study. At each time of analysis ($T_0 - T_6$) samples were tested in duplicate and the mean OD values and percentage remaining activities (% RA) were calculated. PC1: positive control anti-His HRP antibody 1:300 or 1:8,000; PC2: positive control anti-His HRP antibody 1:60,000; NC: negative control; negative samples: 1, 6; positive samples: 11, 12, 3.

Samples	T_0				T_1				T_2				T_3			
	OD 1	OD 2	Mean	% RA	OD 1	OD 2	Mean	% RA	OD 1	OD 2	Mean	% RA	OD 1	OD 2	Mean	% RA
PC1 1:300	2.676	2.687	2.682	100	2.676	2.682	2.679	99.91	2.653	2.655	2.654	98.97	2.646	2.650	2.648	98.75
PC1 1:8,000	2.232	2.250	2.241	100	2.021	2.038	2.030	90.56	1.973	1.988	1.981	88.38	1.875	1.886	1.881	83.91
PC2 1:60,000	3.152	3.123	3.138	100	3.124	3.132	3.128	99.70	3.036	3.042	3.039	96.86	2.953	2.924	2.939	93.66
NC	0.034	0.033	0.034	100	0.030	0.033	0.032	94.03	0.031	0.032	0.032	94.03	0.032	0.030	0.031	92.54
Sample 1 (-)	0.049	0.056	0.053	100	0.045	0.052	0.049	92.38	0.043	0.051	0.047	89.52	0.051	0.040	0.046	86.67
Sample 6 (-)	0.040	0.042	0.041	100	0.038	0.040	0.039	95.12	0.040	0.035	0.038	91.46	0.032	0.034	0.034	82.93
Sample 11 (+)	1.771	1.645	1.708	100	1.772	1.658	1.715	100.41	1.422	1.653	1.538	90.02	1.557	1.477	1.517	88.82
Sample 12 (+)	1.553	1.402	1.478	100	1.571	1.413	1.492	100.98	1.263	1.354	1.354	91.64	1.168	1.202	1.185	80.20
Sample 3 (+)	1.426	1.311	1.369	100	1.438	1.312	1.375	100.47	1.430	1.310	1.370	100.11	1.292	1.175	1.234	90.14

Samples	T_4				T_5				T_6			
	OD 1	OD 2	Mean	% RA	OD 1	OD 2	Mean	% RA	OD 1	OD 2	Mean	% RA
PC1 1:300	2.649	2.612	2.631	98.10	2.615	2.606	2.611	97.35	2.578	2.593	2.586	96.42
PC1 1:8,000	1.861	1.824	1.843	82.22	1.855	1.842	1.849	82.49	1.679	1.754	1.717	76.59
PC2 1:60,000	2.826	2.819	2.823	89.96	2.473	2.449	2.461	78.44	2.237	2.255	2.246	71.59
NC	0.027	0.031	0.029	86.57	0.031	0.029	0.030	89.55	0.028	0.028	0.028	83.58
Sample 1 (-)	0.040	0.043	0.042	79.05	0.042	0.037	0.040	75.24	0.038	0.035	0.037	69.52
Sample 6 (-)	0.034	0.032	0.033	80.49	0.035	0.039	0.037	90.24	0.031	0.031	0.031	75.61
Sample 11 (+)	1.304	1.286	1.295	75.82	1.172	1.311	1.242	72.69	1.210	1.200	1.210	70.84
Sample 12 (+)	1.178	1.134	1.156	78.24	1.090	1.076	1.083	73.30	1.042	1.015	1.029	69.61
Sample 3 (+)	1.189	1.170	1.180	86.19	1.069	0.975	1.022	74.68	1.049	0.949	0.999	73.00

Supplementary Table S11. Real-time stability study. At each time of analysis (T_0 – T_{12}) samples were tested in duplicate and the mean OD values and percentage remaining activities (% RA) were calculated. PC1: positive control anti-His HRP antibody 1:300 or 1:8,000; PC2: positive control anti-His HRP antibody 1:60,000; NC: negative control; negative samples: 1, 6; positive samples: 11, 12, 3.

Samples	T_0				T_1				T_2				T_3				T_4			
	OD1	OD2	Mean	% RA	OD1	OD2	Mean	% RA	OD1	OD2	Mean	% RA	OD1	OD2	Mean	% RA	OD1	OD2	Mean	% RA
PC1 1:300	2.676	2.687	2.682	100	2.684	2.695	2.690	100.30	2.634	2.638	2.636	98.30	2.632	2.635	2.634	98.21	2.630	2.628	2.629	98.04
PC1 1:8,000	2.232	2.250	2.241	100	2.237	2.248	2.243	100.07	2.238	2.251	2.245	100.16	2.239	2.247	2.243	100.09	2.232	2.251	2.242	100.02
PC2 1:60,000	3.152	3.123	3.138	100	3.134	3.129	3.132	99.81	3.124	3.122	3.123	99.54	3.111	3.136	3.124	99.55	3.164	3.167	3.166	100.89
NC	0.034	0.033	0.034	100	0.034	0.033	0.034	100	0.030	0.028	0.029	86.57	0.030	0.031	0.031	91.04	0.030	0.029	0.030	88.06
Sample 1 (-)	0.049	0.056	0.053	100	0.044	0.051	0.048	90.48	0.055	0.035	0.045	85.71	0.051	0.038	0.045	84.76	0.055	0.036	0.046	86.67
Sample 6 (-)	0.040	0.042	0.041	100	0.037	0.040	0.039	93.90	0.037	0.040	0.039	93.90	0.034	0.043	0.039	93.90	0.033	0.037	0.035	85.37
Sample 11 (+)	2.771	2.645	2.708	100	2.866	2.866	2.866	105.84	2.838	2.894	2.866	105.83	2.836	2.892	2.864	105.76	2.839	2.851	2.845	105.06
Sample 12 (+)	2.553	2.402	2.478	100	2.657	2.711	2.684	108.34	2.684	2.586	2.635	106.36	2.680	2.584	2.632	106.24	2.687	2.580	2.634	106.30
Sample 3 (+)	2.426	2.311	2.369	100	2.437	2.515	2.476	104.54	2.534	2.403	2.469	104.22	2.530	2.400	2.465	104.07	2.530	2.409	2.470	104.26

Samples	T_5				T_6				T_7 (3 months)				T_8 (6 months)				T_9 (9 months)			
	OD1	OD2	Mean	% RA	OD1	OD2	Mean	% RA	OD1	OD2	Mean	% RA	OD1	OD2	Mean	% RA	OD1	OD2	Mean	% RA
PC1 1:300	2.616	2.646	2.631	98.12	2.642	2.658	2.650	98.83	2.633	2.647	2.640	98.45	2.650	2.677	2.664	99.33	2.632	2.614	2.623	97.82
PC1 1:8,000	2.250	2.241	2.246	100.20	2.234	2.248	2.241	100.00	2.347	2.378	2.363	105.42	2.238	2.241	2.240	99.93	1.914	1.891	1.903	84.90
PC2 1:60,000	2.902	2.943	2.923	93.15	2.873	2.861	2.867	91.38	2.868	2.871	2.870	91.46	2.712	2.707	2.710	86.36	2.698	2.695	2.697	85.94
NC	0.030	0.028	0.029	86.57	0.031	0.028	0.030	88.06	0.029	0.027	0.028	83.58	0.033	0.026	0.030	88.06	0.030	0.028	0.029	86.57
Sample 1 (-)	0.051	0.035	0.043	81.90	0.056	0.036	0.046	87.62	0.053	0.036	0.045	84.76	0.048	0.037	0.043	80.95	0.040	0.040	0.040	76.19
Sample 6 (-)	0.031	0.036	0.034	81.71	0.035	0.036	0.036	86.59	0.037	0.038	0.038	91.46	0.053	0.033	0.043	104.88	0.042	0.043	0.043	103.66
Sample 11 (+)	2.841	2.881	2.861	105.65	2.843	2.882	2.863	105.71	2.842	2.887	2.865	105.78	2.531	2.530	2.531	93.45	2.196	2.142	2.169	80.10
Sample 12 (+)	2.690	2.601	2.646	106.78	2.690	2.605	2.648	106.86	2.687	2.597	2.642	106.64	2.498	2.525	2.512	101.37	2.009	2.085	2.047	82.62
Sample 3 (+)	2.536	2.414	2.475	104.50	2.534	2.420	2.477	104.58	2.532	2.419	2.476	104.52	2.559	2.523	2.541	107.28	2.063	2.100	2.082	87.88

Samples	T_{10} (12 months)				T_{11} (15 months)				T_{12} (18 months)			
	OD1	OD2	Mean	% RA	OD1	OD2	Mean	% RA	OD1	OD2	Mean	% RA
PC1 1:300	2.628	2.636	2.632	98.15	2.491	2.504	2.498	93.15	2.382	2.391	2.387	89.00
PC1 1:8,000	1.920	1.927	1.924	85.83	1.879	1.882	1.881	83.91	1.653	1.621	1.637	73.05
PC2 1:60,000	2.601	2.603	2.602	82.93	2.471	2.463	2.467	78.63	2.367	2.361	2.364	75.35
NC	0.031	0.028	0.030	88.06	0.030	0.029	0.030	89.20	0.030	0.029	0.029	88.00
Sample 1 (-)	0.046	0.044	0.045	85.71	0.039	0.045	0.042	80.31	0.038	0.043	0.041	77.47
Sample 6 (-)	0.044	0.045	0.045	108.54	0.037	0.039	0.038	93.25	0.035	0.037	0.036	87.49
Sample 11 (+)	2.279	2.202	2.241	82.74	2.247	2.150	2.199	81.20	2.106	1.984	2.045	75.51
Sample 12 (+)	1.884	1.814	1.849	74.63	1.894	1.787	1.841	74.30	1.787	1.681	1.734	70.00
Sample 3 (+)	1.879	1.855	1.867	78.83	1.849	1.763	1.806	76.25	1.795	1.664	1.730	73.02
